# Reference Values on Children’s Hair for 28 Elements (Heavy Metals and Essential Elements) Based on a Pilot Study in a Representative Non-Contaminated Local Area

**DOI:** 10.3390/ijms24098127

**Published:** 2023-05-01

**Authors:** Roberto Ruiz, Carmen Estevan, Jorge Estévez, Carolina Alcaide, Miguel A. Sogorb, Eugenio Vilanova

**Affiliations:** Institute of Bioengineering, Miguel Hernandez University of Elche, 03202 Elche, Spain; rruiz@umh.es (R.R.); cestevan@umh.es (C.E.); jorge.estevez@umh.es (J.E.); msogorb@umh.es (M.A.S.)

**Keywords:** metals, essential elements, biomonitoring, hair, children, reference values

## Abstract

Studies have been published, and laboratories offer services of measuring elements in hair as biomarkers of environmental exposure and/or control of essential elements (trace or macro). These reported values can have only sense if compared with adopted reference values. In this work, we propose provisional reference values based on a pilot child population. The concentrations of 28 elements were measured in children’s hair samples. An observational, descriptive, cross-sectional study was conducted in a typical child population in the Mediterranean region void of excessive pollution problems to analyze 419 hair samples of children aged 3–12 years. Children were selected by a simple random method from eight primary education schools in different municipal districts, which included urban, rural and industrial areas. Samples of around 100 mg were washed and acid digested by an optimized procedure. All measures were performed using ICP-MS with Sc, Y and Re as internal standards. The statistical analysis was performed by two approaches: (a) considering all the data and (b) without outliers (second-order atypical data) to compare them with other published studies. The distribution curves in all the elements studied were asymmetric and did not fit the theoretical normality distributions. Therefore, the analysis based on percentiles was more appropriate. In most elements, only slight differences were observed with sex or age, which did not justify proposing separate reference ranges. From the results of this study, provisional reference values are proposed following two criteria: (a) simple application of the table of percentiles built by removing outlier values and (b) values after a detailed analysis case-by-case, considering other data as the distribution profile and other published data of each element. Although the pilot sample was from a limited area, it was carefully selected to be representative of a general non-contaminated population. With this limitation, the proposed reference values might be useful for researchers and physicians until a wider geographical study is available for a large number of elements.

## 1. Introduction

Harm to health by pollution caused by elements of concern, such as Pb, Hg, Cd, Tl and As, among others, is well-known, and the toxicity of such elements has been extensively studied in recent decades. Interest has been shown in assessing exposure to these elements and their effects on children and their development. The route for exposure might be from ingestion in food [1], drinks [2] or contaminated particles in the air [3] and others. The nomenclature of “trace elements” is frequently used for elements presented in biological samples at low concentrations, either for toxic or biologically essential elements. Essential minerals may be elements encountered in small amounts (Fe, Mn, Cu, I, Co, Zn, F and Se) and so-called macro-minerals, which are required in large amounts (Ca, P, Mg, Na, K and Cl) [4]. 

Some trace elements are considered probably or possibly essential as some beneficial effects have been suggested (i.e., Si and B), and some elements are considered essential by some food agencies but not others, such as Cr. The concentrations of essential minerals in the organism must be balanced, and they may harm health if lacking or found in excess. Some examples are night blindness related to lack of Zn in infant diets, especially in developing countries [5]; lack of Fe, also known as iron deficiency anemia; and problems related with hepatic damage caused by an excess of Cu [6]. They are all frequent problems but rarely harm the health severely. Given this delicate balance, it is important to monitor exposure levels in specific populations and vulnerable age groups, such as children.

Therefore, biomonitoring and assessing incidence on health and on infants’ development is a world concern and a priority for toxic and essential elements. Hair samples have been used as both a non-invasive method and a biomarker of exposure to analyze these elements. Routine methods require between 100 and 150 mg of hair to undergo chemical methods [7]. These samples are ideal for mega-analyses with healthy populations to conduct population studies of interest for public health issues. 

Special efforts have been made to evaluate and biomonitor mercury in infants, and relevant studies have been conducted with Pb and Cd. There is particular concern for areas whose gastronomy is rich in sea products since bioaccumulation capacity may play a role. By focusing on Hg, the series of studies performed with children who live on the Seychelles and Faroe Islands is likely the most widely studied background available [8,9,10,11,12,13,14,15,16,17,18]. Some more limited studies exist, which have been conducted in other geographical areas, such as the Mediterranean, where a great deal of fish and seafood is eaten [19,20]. 

Efforts have been made for larger cohort studies in relationship to neurodevelopment and Hg exposure in Central Europe and Mediterranean countries [19,20]. Other studies have been performed in Japan with a general population, which essentially focused on Hg and other elements, such as Pb or Cd [21,22,23,24] or specifically on a child population [22,23,25,26]. A biomonitoring study in adults in a mining area in Colombia was reported in relationship with renal impairment [27]. Studies focused on children have been reported in Malaysia [28], China [29,30], Brazil [31,32], Poland [33], Czech [34] and Slovenia [35] as well as related to Au mining in Perú [36,37]. Studies relating Hg exposure with autism and other neurological problems are a focus of current attention [38,39,40].

For individual acute-phase studies, known tests that use blood and urine samples can be appropriate. The use of other biological sample, such as autopsy tissues, have been considered, especially for biomonitoring chronic exposure. However, an evaluation in a case study in a population potentially exposed to metals near a hazardous waste incinerator concluded that using autopsy tissues did not show advantages in comparison to other appropriate samples for biomonitoring, such as blood [41].

Yet when studying the problem in large populations, it is necessary to rely on more efficient user-friendly non-invasive biomarkers as far as sampling is concerned. Hair covers all the needed requirements: (1) It is a tissue of an accumulation type that grows 1 cm every month on average. It enables studies to evaluate chronic exposure and allows a temporal relationship to be established in accordance with the hair segment under study. (2) Obtaining hair samples does not involve aggressive invasive methods but merely requires simple techniques with minimum personnel training. (3) No special means or specific care are required to handle, conserve or transport samples. (4) It is a low-cost practice.

Nonetheless, the main problem of human biomonitoring in hair is that reference values are lacking for most elements in the general population, which is stressed even more for children. 

Relevant studies of biomonitoring in hair have been performed with Hg and some with other elements of concern, such as Pb and Cd. However, very few studies have been conducted in multi-element biomonitoring in children’s hair [42,43]. Studies with a limited number of elements have been reported, mainly monitoring elements, such as Pb, Cd, MN, Cr, Fe, Ni, Zn, Al and As in addition to Hg [29,44,45]. 

In some cases, studies are in relationship to smoking, dietary or industrial activity in the area; gender influence; or military attacks [46,47,48,49,50]. More recently, attention has been paid to assess heavy metals in relation to autism [48,49] and other developmental impairment in children and adolescents [51,52,53] as well as the relationship with secondhand cigarette smoke exposure [54], but most studies deal with only a few elements, mainly Hg, Cd, Al, Co, Cu, Ni and Cr. One study in Ethiopia evaluated up to 41 elements in the hair of children. Although this was limited to 81 subjects [55], it is a useful recent study.

For this work, a pilot child population in the Mediterranean was selected. The study area was the municipality of Elche, in the province of Alicante (east Spain), which encompasses urban, rural, farming and industrial areas. The industrial areas neither are heavy industrial areas, nor are they heavily polluted, and this province has no agricultural overproduction areas. Therefore, the population of this area can be considered to be representative of a typical general population of the Mediterranean region without special heavy contamination, and, in this way, we were able to apply a viable logistic to obtain a sufficient statistical number of subjects and an appropriate distribution inside the population.

An observational, descriptive, cross-sectional study was conducted. The total population, aged under 14 years, was 34,109 individuals of both sexes, according to the census (Table 1), from which a representative sample was obtained of 419 children in the range of 3–12 years old. The concentrations of 28 mineral elements were determined in hair samples using ICP-MS technology. The distribution curves for each element were described, and the descriptive statistics and sex and age distributions were analyzed. From the results of this study, provisional reference values are proposed, which may be used in clinical diagnoses and epidemiological studies by public health professionals using hair element concentrations as biomarkers of environmental exposure.

## 2. Results

### 2.1. General Statistics and Distribution

Hair samples from 419 children were selected following the statistic and inclusion/exclusion criteria described in Material and Methods for the design of the study. Samples were washed, dried, digested and analyzed using ICP-MS for measuring the concentrations of 28 elements based on calibration curves with data from certified standards (Table S1).

The general statistics of the obtained data are presented in Table 2. The measuring unit is micrograms of element per gram of dry hair samples. 

**Figure 1:**
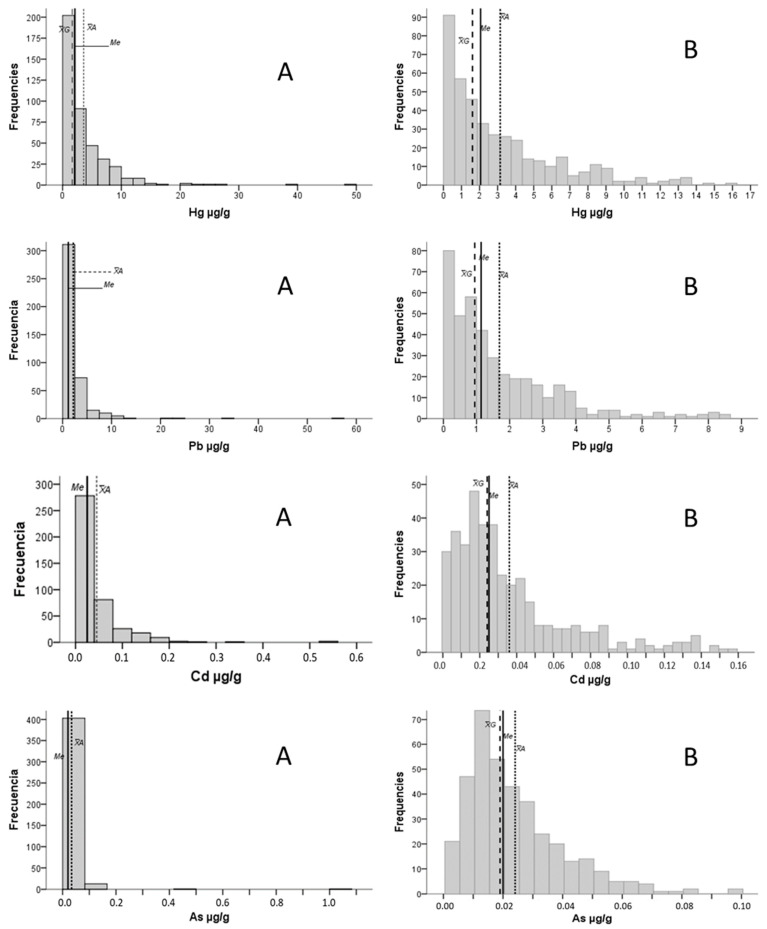
Examples of the distribution curves of several toxic elements. The geometric mean ($\bar{X}$_G_), median and arithmetic mean are indicated by long dashed, dotted and continuous lines. B Panels show distribution splitting in more range categories than A panels, and do not consider extreme values.

**Figure 2:**
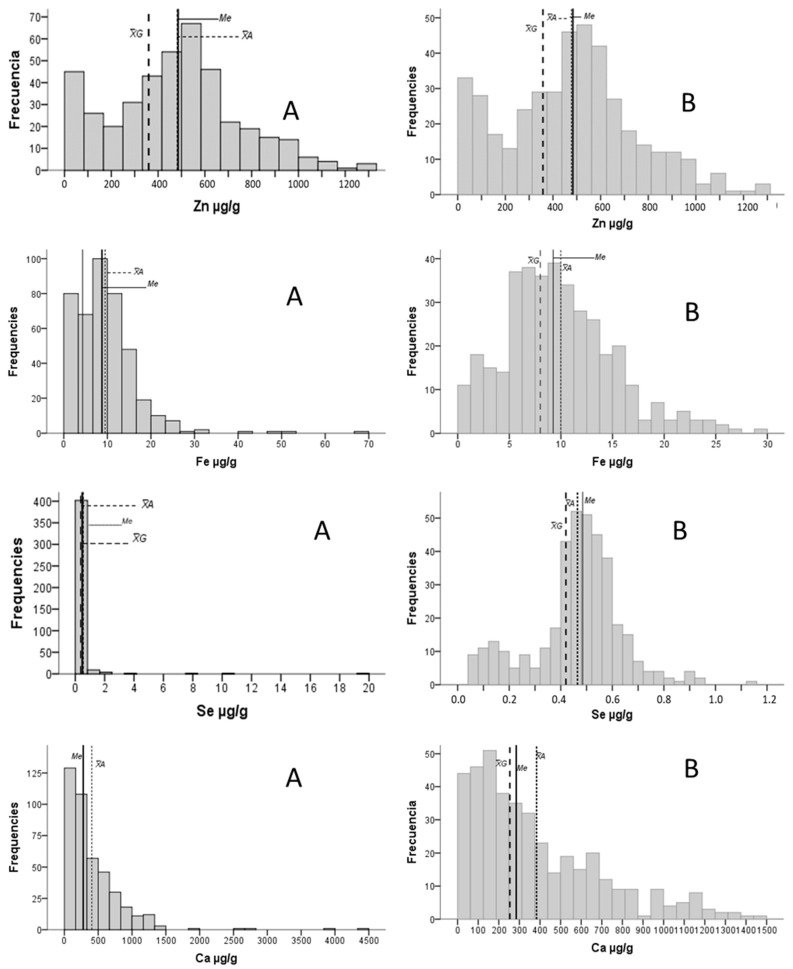
Example of distribution curves of several oligoelements. The geometric mean ($\bar{X}$_G_), median and arithmetic mean are indicated by long dashed, dotted and continuous lines. B Panels show distribution splitting in more range categories than A Panels and do not consider extreme values.

All the analyzed elements presented asymmetric distribution curves (ϒ1 > 0.5) that did not fit the theoretical normality distributions (K-S < 0.05), which makes the mean and standard deviation (SD) weak if used as central tendency and dispersion parameters. All the distribution curves were leptokurtic (ϒ2 > 0.5), and none were mesokurtic. Zn and Al were the elements whose kurtosis coefficients came closest to zero: ϒ2 = 2, for both. By way of example, the heavy metals Hg, Pb, Cd and As are shown in Figure 1. As examples, the most symmetric curves found (Zn, Fe and Se) are presented in Figure 2.

The proportion of the SD values vs. the $\bar{X}$A values of the elements was high, with %SD that exceeded 50%. The variability was very high. The elements with the lowest %SD were Zn and Fe (Zn: 9.48 ± 7.34 µg/g; and Fe: 483.4 ± 291.8 µg/g), with 60% and 77%, respectively. In certain cases (i.e., As) the SD tripled the mean value. This broadness in the ranges of concentrations occurred with most elements, except for U, Be, Ag and Tl, whose scarcity in the samples did not enable suitable observations to be made. Thus, it was considered more suitable to base the data analysis on calculating the percentiles. Such percentile-based analyses considering all data are shown in Table 3 and might be considered for proposing reference values based on the 5–95th percentiles. 

However, due to the high asymmetry of the distribution and the existence of very extreme atypical values, approaches considering eliminating such values were considered as follows.

### 2.2. Analysis by Eliminating Extreme Values

The quartiles technique was used to determine criteria to select the limits of the extreme values (see the criteria in the Section 4). Both degrees of atypicality were calculated by excess: first and second degrees (Table S2). As the calculated representative population sample had to contain 380 individuals, to maintain N above this figure, we selected the second-degree value (excess) as the limit of atypicality. All the values over this limit were considered atypical.

Table S3 shows the general statistics after eliminating the atypical second-degree (excess) values and the values below the LOD. The distribution curves of essential elements, such as Zn and Se, became more symmetric with asymmetry indices close to zero (Zn: ϒ1 = 0.4; and Se: ϒ1 = −0.3. When considering the example of Fe, despite being asymmetric (ϒ1 = 0.9), the contrast was evident when comparing the A panels with the B panels in Figure 2. The asymmetric distribution remained in the heavy metals, even after eliminating the atypical values (ϒ1 > 0.5).

This makes the mean and the SD weak as central tendency and dispersion measures. Therefore, we calculated a table of the values of the percentiles after eliminating the atypical values and those below the LOD (Table S4). 

### 2.3. Crossing of Age and Sex Variables 

The distribution of the elements in the study population did not meet the normality assumption. Therefore, for the crossing of variables, non-parametric tests were applied: Kruskal–Wallis for age and the Mann–Whitney U test for sex.

#### 2.3.1. Age and Study Elements

The ages of the children in years were grouped into three mutually exclusive 3-year class intervals: 3–6 years (*n* = 156); 7–9 years (*n* = 162); and 10–12 years (*n* = 101). Differences were found among these groups in the concentrations of the following elements for age: Be, Mg, Al, K, Ca, Mn, Co, Cu, Zn, As, Se, Sr, Ba, Bi, Tl, Ag, Au and U (*p* < 0.01) (Table 4). 

The age variable was processed as ordinal categorical and numerical. As a numerical variable, the degree of the correlation found with the study elements was measured using Spearman’s Rho coefficient, and correlation coefficients were found within a range from −0.266 (As was the most negative) to 0.311 (Sr was the most positive). 

In all the elements for which a correlation with age was found, with both a falling and rising tendency, the correlation coefficients were <0.5, that is, the correlations were weak, scarce or null, which means that, although they were observed, they were not strong enough to explain the upward/downward tendency of an element according to age. Moreover, this tendency was influenced by other variables. 

#### 2.3.2. Sex and Study Elements

Differences in the concentrations were observed as higher in boys for B, Na, K, Zn and As but were higher in girls for Mg, Ca, Co, Ni, Cu, Sr and Ba (*p* < 0.01) with no differences for Al, Cr, Mn, Fe, Se, Mo, Cd, Hg, Pb and Bi (Table 5). 

From the results of this study, we considered that the differences by age and sex were not sufficient to justify separate analysis for proposing reference values inside the child population.

## 3. Discussion

The main purpose of this paper is to propose reference values for concentrations of elements in the hair of children. We based this work on the statistical data obtained from the hair samples of a representative population in the Mediterranean region that were analyzed to obtain concentrations of heavy metals and essential elements by ICP-MS technology. 

The distribution curves per element were described, and the descriptive statistics and the sex and age distributions were analyzed to propose the references values, which are presented in this discussion. Attempts to establish reference values of a wide number of multiple elements in hair have been published regarding university students in Poland [56] and schoolboys in Rome [40], and an interesting review was published [57].

### 3.1. Statistical Considerations

In many of the published studies, especially on Hg, authors have eliminated extreme values in order to reduce the asymmetry of the distribution as much as possible, with the goal of better applying the parametric statistical calculations.

We considered it appropriate to include all data obtained from the sample of the population and, therefore, to use non-parametric statistics, which is recommended for the analysis of data not adjusted to a theoretical normal distribution (see the general statistics in Table 2 and percentiles in Table 3). Nevertheless, to compare our data with those published by other authors, in this work, we also show descriptive statistical analyses after eliminating extreme (excess) and below the LOD (zero) values (Table S3) and the corresponding table of percentiles (Table S4).

Even doing this last, the asymmetry is maintained, so non-parametric statistics continue to be the technique of choice, and the mean and standard deviation lose strength. Therefore, we proposed reference values based on the percentiles.

### 3.2. Sex and Age Variables

While it is true that differences were found in the concentrations of several elements in relation to the age and sex variables (Table 4 and Table 5) and that these differences fit with those found by other authors in related works [42,43] we consider that they are not sufficient to justify proposing tables of references values of for each age and sex group.

The margins of reference that we propose are in a sufficiently wide range to include the entire study population in the same table. For practical purposes, we propose a single table of references, without different margins in relation to age and sex.

### 3.3. Criteria to Propose Reference Values for Elements in Hair of Children: Criteria

The main objective of this work was to propose a provisional tentative table of reference values for the concentrations of heavy metals and essential elements using hair as the matrix analysis of the biomarkers of exposure. Selecting these reference margins was based on statistical analysis without considering clinical criteria. To this end, a representative population sample was selected: school children. We studied two approaches to obtain reference values:

(1) First proposal: Based on the statistics of the data presented herein, mainly using 5–95th percentiles after eliminating outliers (Table S4) or in some cases from the percentile table considering the total population of study (Table 3). The distribution curves of all the studied elements were completely asymmetric, and none fitted theoretical normality. Hence, the means and standard deviations had a low statistical value, and the percentiles were considered more suitable. Although the 5–95% range of percentiles might be the recommendable criteria, professionals can use other ranges that they consider to be more suitable in each case.
(2) Second proposal: Setting values for the specific analysis of each element by considering, additionally to the percentile table, other data as follows: (i) the characteristics of the statistical distribution shown with and without eliminating extreme outliers and (ii) comparing our results in published works with a similar population in a case-by-case approach. The intention here was for them to be of general potential use. A descriptive analysis of the criteria applied for each element is shown in Supplementary Table S5. The main bibliographic references used to apply such criteria are shown inside that table.

The proposed values are summarized in Table 6 for both criteria.

## 4. Materials and Methods

### 4.1. Design of Study

#### 4.1.1. Type of Study, Place and Time

An observational, descriptive, cross-sectional study was conducted in the municipality of Elche, located in the province of Alicante, east Spain. Selection of study subjects: a simple random method; time: between September and December 2006.

#### 4.1.2. The Total Study Population and Population Sample

The population data, obtained from the Spanish National Institute of Statistics (INE), were tabulated by age groups in mutually exclusive 4-year intervals. The total population, aged under 14 years, constituted the total study population in the municipality of Elche. 

There were 34,109 individuals of both sexes, according to the census updated in September 2006, with this being the field sampling starting time. The data are shown in Table 1. The population sample was selected from elementary school children under the age of 12 years old. Conformity of Ethical Committee of University Miguel Hernandez (reference: 2017.167.E.OEP, Ref.: IB.EVG.02.17).

Sample calculation: The sample was calculated using the statistical formula recommended for finite populations: *n* = N*Z^2^α*p*q/d^2^*(N − 1) + Z^2^α*p*, where N = 34,109 was the total study population of children in the area. A confidence level of 95% (which corresponds with a preset value of Zα = 1.96) was considered. The prevalence of the problem was unknown at the time; therefore, the ratio value was also unknown. Hence, we used *p* = 0.5 (50%) to maximize the sample size; q = 1–9 = 0.5, and the standard precision or acceptable limit error of the sample was 5% (d = 0.05). On this basis, a value of *n* = 380 individual was estimated. Increasing the sample size by 5% is usually recommended for dealing with any unforeseen absence of responses, records, processing errors, samples that are not useful, etc. In our study, the final increase in samples for contingencies was 10%.

The guest sample was made of 1200 individuals preselected to be invited to participate. From them, the collected sample were 422 individuals attempting to be representative of the different subareas and age–sex distribution. Finally, we had 419 useful samples producing data.

Inclusion criteria: children aged 3–12 years; both sexes; residents in the municipality of Elche; any ethnic group; not undergoing any hair treatments (dyes, treatments to avoid hair falling out, revitalizing products or similar) in the 3 months prior to sample collection; with sufficient hair length to obtain a useful sample; and their parents had signed the informed consent.

Sampling method: probabilistic, randomly simple. Schools were selected within each school district. The cooperation of the local Education Authorities was fundamental in this phase of the work. The project was presented to the Students–Parents Association, school directors and teachers. The distribution of the data collection instrument (survey and informed consent) for the guest sample was performed randomly. The selection of subjects was conducted on site, randomly from among those who attended on the day of sample collection and from among those whose parents had provided authorized written permission to participate in the study.

Sample collection: 419 useful samples were collected in eight schools, one school per district. The weight and height were measured, and the age and sex were recorded. A hair sample was obtained by cutting a small section of hair from the occipital area, close to the scalp. Each sample was kept in a self-closing plastic bag and was coded.

#### 4.1.3. Study Variables

Sex: male and female; Age groups: 3–6; 7–9; and 10–12 years; Elements (µg/g in dry hair): Be, B, Na, Mg, Al, K, Ca, V, Cr, Mn, Fe, Co, Ni, Cu, Zn, As, Se, Sr, Mo, Cd, Ba, Hg, Tl, Pb, Bi, Au, Ag and U.

### 4.2. Analytical Procedures

#### 4.2.1. Hair Sample Processing

Hair samples (0.1–0.3 g each) were immersed in 200 mL Triton X 100 at 2% for 1 min in an ultrasound bath. They were rinsed three times with ultrapure water, each time for 1 min. Hair was dried for 24 h at 60·°C. Hair samples were cut with surgical steel scissors into ≤1 mm pieces to facilitate digestion. Scissors were previously treated with 0.6% nitric acid for 10 min. Approximately 0.1 g of sample was placed into pre-weighted Falcon tubes, and the exact weights were noted. Then, they were centrifuged at 5000 rpm for 5 min for moving hair pieces to the bottom of the tube. Next, 1 mL of HNO_3_ 65% Suprapur^®^ was added, and the samples were left in an acid digestion for 48 h. 

After digestion, they were transferred to clean tubes, and 9 mL of ultrapure water was added to adjust to 10 mL to give a nitric acid concentration of up to 6.5%. Finally, 5 mL of each sample was placed in previously prepared tubes with 0.5 mL of the internal standard mix to obtain a final volume of 5.5 mL in each tube. The remaining 5 mL was stored as backup.

Several washing procedures were previously checked, and a decision was made to apply an intensive (ultrasound and detergent) but short (1 min) treatment to eliminate superficial materials and to avoid removing the very soluble ions, such as Na and Mg, from inside the hair as these are not tightly fixed to the hair tissue.

#### 4.2.2. ICP MS Measures

The equipment used was an Inductively Coupled Plasma Mass Spectrometer (ICP-MS), Agilent^®^ Model 7500A (Agilent Technologies Spain S.L., Madrid, Spain). The sampling pump was programmed to 1 mL/min, 30 s for washing tubing, 30 s for stabilizing detection and then 3 s by measuring per mass in duplicate (total time of around 2 min per sample). Approximately 160 samples can be measured in 8 h. Therefore, the 419 hair samples and calibration standard solutions were measured in three batches. For each batch, the procedure was repeated: first, the ICP-MS and TUNE standards were measured, followed by hair samples and blank controls. A standard was introduced every 20 samples.

#### 4.2.3. ICP-MS Certified Standards and Calibration

The certified standard solution used for calibrating the equipment and for the semiquantitative analysis was Agilent Technologies Tuning solution 10 µg/L Li, Y, Ce, Tl, Co (Agilent Technologies Spain S.L., Madrid, Spain)). Sc (m/z 45), Y (m/z 89) and Re (m/z 185) ICP standards 1000 mg/L, CertiPUR^®^ (Merck, Darmstadt, Germany)), were used as internal standards (ISTD). For this purpose, a mix solution was prepared that contained the three elements at (100 µg/L in 5% HNO_3_). A 0.5 mL volume of the mix was added to 5 mL of each sample or to a calibration standard.

Calibration standard for the full quantitative data analysis. Twelve standard solutions were prepared by serial dilution (ratio 1:2) from CertiPUR^®^ Merck ICP multi-element standard solution X (23 elements in nitric acid) and the mercury ICP/MS standard by dilution with nitric acid 65% Suprapur^®^ (Merck). They were used for the calibration curves of each element under study Table S1. For Cu and Zn, three other standards at higher concentrations were prepared using zinc sulfate 7-hydrate PANREAC^®^ and copper sulfate pentahydrate PANREAC^®^ (Panreac Química S.L.U., Barcelona, Spain) because the concentrations of these elements in many hair samples exceeded the ICP multi-element standard solution concentration. To 5 mL of each calibration standard solution, a 0.5 mL volume of the mix of the internal standards was added formed by a solution containing 100 µg/L of Sc, Y and Re (CertiPUR^®^, Merck) in 5% HNO_3_).

Calibration curves. Calibration curves were obtained by the mathematical function equation that best conformed to the calibration curve points within the range of concentrations within which all the elements were found in the hair samples.

Laboratory blank control and standard control. Three blank controls were made by each set of analyzed samples and were subjected to the same process as the hair samples. For checking the equipment variability over time, the Tuning solution and a calibration solution were measured as a control every 20 samples.

Validation Reference Materials. Validation of the methodology was performed testing with several reference materials: IAEA-086 (un-spiked), obtained from International Atomic Energy, Analytical Quality Control Service, Vienna, Austria; NIES No. 13 obtained from National Institute for Environmental Studies as Certified Reference Material No. 13 “Human Hair”; and ERM-DB001 obtained from European Reference material, Join Research Center, Institute for Reference Materials and Measurements).

Data analysis. The counts obtained for elements Be, B, Na, Mg, Al, K, Ca, V, Cr, Mn, Fe, Co, Ni, Cu, Zn, As, Se, Sr, Mo, Cd, Ba, Hg, Tl, Pb and Bi were analyzed by the full quantitative method included in the equipment’s software. For this purpose, the signals, corrected by the internal standard, were compared with the calibration curves deduced in the standard solutions per element. However, for Ag, Au and U, the semiquantitative analysis option of the equipment’s software was used based on the calibrations performed with the aforementioned tune solutions. The data obtained with the ICP-MS computer software were copied into an an electronic sheet to transform the units mathematically from µg/L into µg/g of dry hair according to the weight of each sample. Data were copied to the SSPS software (IBM-SPSS Statistics 18-22) to be statistically analyzed.

### 4.3. Statistical Approaches

Asymmetry indices and normality test. The Fisher asymmetry coefficient (ϒ1) and kurtosis coefficient (ϒ2) for each element were calculated to observe the behavior of the distribution curves. The K-S normality test was applied to check if the data distribution in the study population met the normality assumption.

Estimation of central tendency and dispersion. Several central tendency and dispersion parameters were calculated as follows: arithmetic mean ($\bar{X}$A) with its respective 95% confidence intervals; standard deviation (SD); median (Me); range of concentrations (minimum–maximum); and percentile values 5%, 10%, 15%, 25%, 50%, 65%, 75%, 85%, 90% and 95%.

To calculate the geometric mean ($\bar{X}$G) and to compare our data with those published by other authors, the values that equaled zero (below the limit of detection, LOD) were removed and excluded from the calculations.

Data tabulation: Data were grouped into tables per categories, calculated using the formula of Sturges: class interval = range/K, where the K value = 1 + 3.322 (Log 10N), where K is the number of categories and N is the total study population. Histograms were constructed according to these tables.

Outlier values: The elements distribution in the study population did not meet the normality assumption (see Section 2.1). For this reason, the standard deviation lost strength as a statistical parameter of dispersion in our data. Therefore, the quartiles technique was used to identify the upper boundary outliers, both first-degree and second-degree. For that, the value of the interquartile range (IQR) was calculated by the difference between the third quartile (Q3) and the first one (Q1): IQR = Q3 − Q1. The limits of atypical values, were obtained as follows:

First-degree limit of atypical value:

Excess: >Q3 + (IQR × 1.5).

Second-degree limit of atypical value:

Excess: >Q3 + (IQR × 3).

## 5. Conclusions

Several studies have been reported that have used measures of biomarkers in hair, especially with Hg and as well as studies with heavy metals, such as Pb and Cd. Very few studies have been published with multi-elemental values in children’s hair. Special consideration is needed for Hg. The Mediterranean population is a high fish and shellfish consumer, which explains the higher level compared with other populations. Therefore, the value based on the 95th percentile for Hg is likely only representative for similar high-fish-consuming populations.

For all elements, the proposed value suggested with the second criteria takes this factor into consideration for suggesting a value for a more general potential application. The data and arguments supporting this are discussed in Table S5, case by case, for each element. The proposed reference values for the concentrations of elements in the hair of children must be cautiously considered as provisional until obtaining values obtained with more universal geographical distribution studies and multi-center-based data. Nevertheless, the area of study and the sample were carefully selected to be representative of the general child population, including individuals in in different rural, urban and industrial areas and with, in each subarea (school), a careful distribution of age, sex and other random characteristics, including children of different social, economic, cultural and ethnic conditions. 

Therefore, we consider that the sample should be very similar to a general child population of the whole country and likely also to other similar Mediterranean countries. Our approach allowed us to apply viable logistics to obtain an appropriate sample. Moreover, the values proposed with the second criteria consider other data that allow us to consider it reasonable to be applied provisionally for other wider populations.

In any case, the reference proposed in Table 6 for concentrations of elements in hair of children should be useful as a reference for biomarkers of exposure by environmental health professionals and epidemiologists at least for preliminary references for comparison of data of individuals or groups.

## Figures and Tables

**Table 1:** Child population according to sex and age (years old).

	0–4	05–09	10–14	Total
Male	6175	5463	5848	17,486
Female	5859	5247	5517	16,623
				34,109

Source: Spanish National Institute of Statistics (INE). The population is indicated in 5-year groups in the 2006 review of the municipal census of Elche.

**Table 2:** General statistics (*n* = 419). Values are expressed as µg/g of hair.

Elem.	m/z	LOD	Min–Max	$\bar{X}$_A_ ± SD	CI 95%	Me	ϒ_1_	ϒ_2_	K-S (*p*)
Be	9	0.0003	0.000–0.013	0.001 ± 0.002	0.001–0.001	0.000	4	18	<0.01
B	11	0.08	0.00–15.80	2.51 ± 2.03	2.31–2.70	2.33	2	8	<0.01
Na	23	2.8	0.0–1077	142.7 ± 148.7	128–157	97.7	2	8	<0.01
Mg	24	0.13	0.70–355.2	28.7 ± 29.8	25.9–31.6	18.7	4	34	<0.01
Al	27	0.39	0.00–43.39	7.68 ± 7.80	6.93–8.43	5.98	1	2	<0.01
K	39	0.56	0.00–315.1	34.5 ± 40.9	30.6–38.5	21	3	9	<0.01
Ca	43	0.8	0.000–4366	406 ± 446	363–449	278.9	4	25	<0.01
V	51	0.0004	0.000–6.054	0.13 ± 0.346	0.097–0.163	0.077	14	219	<0.01
Cr	53	0.005	0.000–17.56	0.215 ± 0.978	0.121–0.309	0.135	15	248	<0.01
Mn	55	0.004	0.000–1.139	0.159 ± 0.145	0.15–0.17	0.119	2	8	<0.01
Fe	56	1.22	0.000–69.114	9.48 ± 7.34	8.7–10.2	8.78	3	14	<0.01
Co	59	0.001	0.000–0.754	0.016 ± 0.054	0.011–0.022	0.006	10	118	<0.01
Ni	60	0.005	0.000–3.4	0.278 ± 0.345	0.245–0.311	0.216	5	31	<0.01
Cu	63	0.05	0.700–863.6	105.7 ± 126.5	94–118	67.2	3	9	<0.01
Zn	66	0.13	10.400–1882	483.4 ± 291.8	455–511	485.5	1	2	<0.01
As	75	0.003	0.000–2.142	0.034 ± 0.118	0.022–0.045	0.02	15	254	<0.01
Se	82	0.05	0.020–19.51	0.55 ± 1.16	0.43–0.66	0.48	13	189	<0.01
Sr	88	0.02	0.000–50.198	4.68 ± 4.73	4.23–5.14	3.14	3	20	<0.01
Mo	95	0.001	0.000–1.174	0.051 ± 0.09	0.043–0.060	0.039	10	111	<0.01
Cd	107	0.002	0.000–1.064	0.045 ± 0.075	0.038–0.052	0.025	8	90	<0.01
Ba	111	0.002	0.000–3.257	0.355 ± 0.328	0.32–0.39	0.289	3	17	<0.01
Hg	137	0.003	0.014–48.122	3.58 ± 4.77	3.13–4.04	2.09	4	27	<0.01
Tl	205	0.0001	0.000–0.006	0.0003 ± 0.001	0.0002–0.0003	0.000	4	25	<0.01
Pb	202	0.03	0.000–55.313	2.19 ± 4	1.81–2.58	1.17	8	87	<0.01
Bi	205	0.0001	0.000–1.994	0.032 ± 0.152	0.017–0.046	0.005	9	101	<0.01
Ag	208	0.003	0.000–0.038	0.001 ± 0.003	0.001–0.001	0	8	76	<0.01
Au	209	0.0002	0.000–1.27	0.029 ± 0.118	0.018–0.040	0	7	58	<0.01
U	238	---	---	---	---	--	--	--	---

LOD: limit of detection; $\bar{X}$_A_: arithmetic mean; $\bar{X}$_G:_ geometric mean; SD: standard deviations; Me: median. ϒ_1:_ asymmetry; ϒ_2:_ kurtosis; and KS: Kolmogorov–Smirnov test.

**Table 3:** Percentiles in the entire study population (*n* = 419). Values as µg/g of hair.

	5	10	15	25	50	65	75	85	90	95
Be	0.000	0.000	0.000	0.000	0.000	0.000	0.001	0.002	0.002	0.004
B	0.00	0.08	0.19	1.17	2.33	2.86	3.59	4.31	4.89	6.11
Na	6.0	12.1	22.3	44.2	97.7	146.3	189.3	249.1	334.7	439.6
Mg	2.5	4.2	5.9	8.6	18.7	31.2	39.9	56.3	67.8	81.9
Al	0.00	0.00	0.22	0.9	5.98	8.81	11.94	14.5	18.83	24.78
K	0.2	1.8	3.6	7.3	21	33.2	45.3	66	82.3	114.4
Ca	23.8	42.3	71.3	127.4	278.9	422	567	723.1	863.6	1133.8
V	0.007	0.014	0.02	0.032	0.077	0.118	0.151	0.196	0.237	0.314
Cr	0.00	0.00	0.00	0.024	0.135	0.177	0.199	0.242	0.279	0.357
Mn	0.006	0.017	0.032	0.067	0.119	0.163	0.211	0.292	0.345	0.45
Fe	0.42	1.08	1.97	5.4	8.78	10.85	12.72	15.1	16.9	21.27
Co	0.00	0.00	0.00	0.00	0.006	0.012	0.016	0.023	0.028	0.041
Ni	0.000	0.015	0.023	0.062	0.216	0.286	0.365	0.468	0.559	0.714
Cu	3.4	5.4	7.6	16.2	67.2	98.8	142.2	201.7	266	371.1
Zn	39.5	77.5	131.9	285.9	485.5	568.5	634.6	759.9	851.1	978.3
As	0.002	0.004	0.007	0.011	0.02	0.026	0.033	0.043	0.052	0.068
Se	0.04	0.08	0.14	0.36	0.48	0.53	0.56	0.61	0.65	0.79
Sr	0.3	0.5	0.69	1.24	3.14	5.31	7	9.13	10.99	13.37
Mo	0.002	0.005	0.007	0.02	0.039	0.052	0.063	0.077	0.09	0.111
Cd	0.002	0.004	0.007	0.013	0.025	0.038	0.049	0.078	0.1	0.146
Ba	0.021	0.036	0.056	0.12	0.289	0.407	0.499	0.645	0.713	0.901
Hg	0.08	0.19	0.32	0.74	2.09	3.41	4.67	6.76	8.58	10.98
Tl	0.000	0.000	0.000	0.000	0.000	0.000	0.000	0.001	0.001	0.001
Pb	0.08	0.15	0.21	0.50	1.17	1.92	2.54	3.60	4.33	7.39
Bi	0.000	0.001	0.001	0.002	0.005	0.008	0.012	0.02	0.038	0.088
Ag	0.000	0.000	0.000	0.000	0.000	0.000	0.001	0.001	0.002	0.003
Au	0.000	0.000	0.000	0.000	0.000	0.000	0.000	0.006	0.066	0.171
U	0.000	0.000	0.000	0.000	0.000	0.000	0.000	0.000	0.000	0.000

**Table 4:** Age group, element Kruskal–Wallis test and bivariant correlation (Spearman’s Rho).

	Rank Average (Median/Arithmetic Mean in µg/g)				Bivariant Correlation	
Elem.	Age 3–6 (*n*=156)	Age 7–9 (*n*=162)	Age 10–12 (*n*=101)	*p* Value *	Rho Spearman	p *
Be	194 (0.00/0.00)	213 (0.00/0.001)	231 (0.00/0.001)	0.014	0.123	0.012
B	197 (2.27/2.32)	223 (2.42/2.75)	210 (2.25/2.42)	0.150	0.062	0.207
Na	209 (92/148)	204 (95/136)	221 (102/146)	0.549	0.032	0.519
Mg	178 (14/21)	213 (21/28)	255 (32/41)	0.000	0.284	0.000
Al	234 (8/10)	189 (5/6)	207 (5/7)	0.004	−0.143	0.003
K	231 (29/45)	199 (20/30)	195 (19/26)	0.020	−0.166	0.001
Ca	183 (227/291)	213 (305/430)	247 (409/546)	0.000	0.247	0.000
V	217 (0.08/0.11)	200 (0.07/0.16)	215 (0.09/0.11)	0.391	−0.009	0.849
Cr	211 (0.15/0.24)	205 (0.13/0.24)	216 (0.14/0.14)	0.774	−0.016	0.737
Mn	245 (0.15/0.21)	183 (0.11/0.13)	199 (0.11/0.14)	0.000	−0.209	0.000
Fe	226 (9.7/10)	195 (8.2/9)	210 (8.2/10)	0.083	−0.089	0.069
Co	215 (0.008/0.019)	193 (0.005/0.016)	231 (0.008/0.014)	0.036	0.018	0.720
Ni	196 (0.19/0.25)	210 (0.22/0.30)	232 (0.25/0.28)	0.063	0.109	0.026
Cu	180 (52/74)	210 (65/114)	256 (126/142)	0.000	0.239	0.000
Zn	168 (389/391)	227 (529/524)	248 (567/561)	0.000	0.275	0.000
As	246 (0.024/0.035)	200 (0.019/0.042)	171 (0.015/0.018)	0.000	−0.266	0.000
Se	184 (0.45/0.52)	221 (0.49/0.60)	233 (0.51/0.50)	0.002	0.173	0.000
Sr	175 (2.32/3.41)	214 (3.82/4.72)	257 (5.44/6.59)	0.000	0.311	0.000
Mo	225 (0.04/0.06)	204 (0.04/0.05)	196 (0.04/0.04)	0.128	−0.086	0.079
Cd	218 (0.03/0.04)	203 (0.02/0.04)	210 (0.03/0.06)	0.542	−0.048	0.330
Ba	190 (0.24/0.31)	209 (0.29/035)	243 (0.36/0.44)	0.003	0.197	0.000
Hg	212 (2.10/3.70)	199 (1.79/2.98)	225 (2.46/4.36)	0.219	0.034	0.489
Pb	223 (1.36/2.54)	194 (1.08/1.56)	215 (1.21/2.67)	0.101	−0.061	0.215
Bi	244 (0.007/0.037)	193 (0.004/0.035)	185 (0.003/0.018)	0.000	−0.245	0.000
Tl	203 (0.0002/0.000)	218 (0.0003/0.000)	207 (0.0002/0.000)	0.30	0.024	0.629
Ag	225 (0.001/0.000)	202 (0.001/0.000)	200 (0.001/0.000)	0.04	−0.135	0.006
Au	201 (0.0160.000)	227 (0.00330.000)	196 (0.00420.000)	0.01	0.083	0.090
U	210 (0.000/0.000)	210 (0.000/0.000)	209 (0.000/0.000)	0.73	−0.038	0.442

* p value, level of significance < 0.05.

**Table 5:** Elements according to sex: Mann–Whitney U test. Rank average (median/arithmetic mean in µg/g).

Elem.	Male (*n* = 202)	Female (*n* = 217)	p *
Be	202 (0.001/0.000)	214 (0.001/0.000)	0.440
B	230 (2.5/2.8)	191 (2.0/2.3)	0.001
Na	230 (110/165)	191 (82/122)	0.001
Mg	186 (16/24)	232 (26/33)	0.000
Al	199 (5.5/7.1)	221 (6.4/8.2)	0.062
K	237 (28/41)	185 (17/29)	0.000
Ca	186 (243/326)	232 (345/481)	0.000
V	181 (0.05/0.09)	237 (0.11/0.17)	0.000
Cr	212 (0.14/0.29)	208 (0.13/0.14)	0.727
Mn	218 (0.13/0.17)	203 (0.12/0.15)	0.204
Fe	214 (8.8/9.7)	206 (8.8/9.9)	0.534
Co	185 (0.004/0.014)	234 (0.010/0.018)	0.000
Ni	196 (0.18/0.24)	223 (0.24/0.31)	0.026
Cu	192 (57/87)	227 (89/123)	0.003
Zn	227 (528/527)	194 (462/443)	0.005
As	240 (0.024/0.030)	182 (0.015/0.037)	0.000
Se	213 (0.47/0.48)	207 (0.48/0.60)	0.595
Sr	184 (2.4/3.8)	234 (4.4/5.5)	0.000
Mo	199 (0.04/0.05)	220 (0.04/0.06)	0.068
Cd	209 (0.025/0.046)	211 (0.026/0.044)	0.823
Ba	194 (0.24/0.32)	225 (0.33/0.39)	0.007
Hg	215 (2.1/3.8)	205 (2.1/3.4)	0.392
Pb	210 (1.15/2.42)	210 (1.17/1.98)	0.959
Bi	213 (0.005/0.024)	207 (0.005/0.038)	0.609
Tl	220 (0.000/0.000)	201 (0.000/0.000)	0.021
Ag	193 (0.001/0.000)	226 (0.001/0.000)	0.000
Au	211 (0.031/2.094)	209 (0.027/0.000)	0.857
U	209 (0.000/0.000)	211 (0.000/0.000)	0.172

* p value, level of significance < 0.05.

**Table 6:** Proposal of reference values. The proposed values are indicated as a limit of maximum value or a range (minimum–maximum) for the essential elements. Values are expressed as µg element/g hair, equivalent to mg/kg as indicated by some authors. Data in the first column are values based on the simple criteria of 5–95th percentiles, eliminating the outliers’ values. The proposed values in the right column (in bold) are modulated considering, case by case, the distribution in our statistics and the other published data as discussed in Supplementary Table S5.

	Based on 5–95 PC (µg/g)	Proposal Considering Other Data (µg/g)
Be	0–0.004	**<0.01**
B	0.2–6.1	**<6**
Na	8–440	**3–600**
Mg	0.4–26	**4–100**
Al	0.6–26	**<20**
K	2–92	**2–200**
Ca	35–1100	**20–1000**
V	0.01–0.28	**<0.4**
Cr	0.01–0.35	**<0.4**
Mn	0.01–0.4	**0.03–1.1**
Fe	2–21	**2–20**
Co	0.001–0.04	**<0.04**
Ni	0.02–0.7	**<0.70**
Cu	3–330	**5–100**
Zn	39–970	**40–850**
As	0.01–0.06	**<0.1**
Se	0.1–0.8	**0.1–1**
Sr	0.3–13	**<5**
Mo	0.01–0.1	**<0.1**
Cd	0.003–0.11	**<0.1**
Ba	0.02–0.9	**<0.9**
Hg	0.08–9	**<3**
Tl	0–0.001	**<0.001**
Pb	0.09–5.2	**<2**
Bi	0.001–0.02	**<0.03**
Ag	0–0.01	**<0.01**
Au	0–0.2	**<0.2**
U	<0.001	**<0.001**

## Data Availability

Not applicable.

## Supplementary Materials

The following supporting information can be downloaded at: https://www.mdpi.com/article/10.3390/ijms24098127/s1. References [58,59,60,61,62,63,64,65,66,67,68,69,70,71,72,73,74,75,76,77,78,79,80,81,82] are cited in the Supplementary Materials
