# Supplementary material for: Reference Values on Children’s Hair for 28 Elements (Heavy Metals and Essential Elements) Based on a Pilot Study in a Representative Non-Contaminated Local Area
Source: Int J Mol Sci. 2023 May 1;24(9):8127. doi: 10.3390/ijms24098127 (PMC10179086; doi:10.3390/ijms24098127)
Supplement: Supplementary file 1 [file ijms-24-08127-s001.zip › Table S5.pdf]

Table S5. Proposal of reference values with Discusion of criteria. The proposed limit of maximum value or a range (minimum-maximum) is indicated for the oligoelements. Values expressed as µg element/g hair and ordered by the atomic mass used for measuring them by ICP-MS

The simplest approach might be to use the 5-95 percentiles as criteria for proposing reference values. However, in that case, they only could be applied to similar populations. To make it possible to apply to a more general population, they have been revised considering the following factors:

(i).-The specific data of the statistical distribution of each element, either with or without eliminating extreme ouliers (critical data summarized in 3rd column.

(ii)-Comparison with publications that reported data from other populations.

Several studies have been reported that have used measures of biomarkers in hair, especially with Hg, and some studies with heavy metals like Pb and Cd. Very few studies have been published with multi-elemental values in children's hair. The proposed values and significant studies are summarized in 4th column and discussed in the right column.

Element	Proposed Values (µg/g)	Data considered from this study	Summary relationship with published data	DISCUSSION
Be	**< 0.01µg/g**	95th percentile 95 = 0.004 µg/g.<br>Max 0.013 µg/g.	Lower one-order of magnitude than healthy controls in Kuwaiti children with autism [58]	*Berilium.* Fido & Al-Saad (2005) [58] conducted a case-control study in children with autism in Kuwait. They reported levels with a higher order of magnitude to those in our population, where 95% had less than 0.004 µg/g and the maximum concentration was 0.013 µg/g. We propose the maximum value, rounded off to **0.01 µg/g**, as the maximum value, which includes 100% of the population. No minimum value is proposed.
B	**<6 µg/g**	95th percentile = 6.11 µg/g similar if outliers were excluded.<br>Below atypical 1st and 2nd degrees (7.22 and 10.85 µg/g)	No data.	*Boron.* The 95th percentile with both n=419 and n= 376, after removing outliers, are practically equal and are below the atypical values, excess for the 1st and 2nd degrees. No studies that compared to ours were found. We suggest using the 95th percentile, rounded off to **6 µg/g**, as the maximum reference value.
Na	**3–600 µg/g**	95th percentile =440 µg/g (429 µg/g if outliers were excluded).<br>Atypical 2nd degree =625 µg/g.<br>Minimum excluding LOD=3 µg/g.<br>Including 98.7% of our population.	5-fold higher than S Korean children [42].<br>1/3 lower than Polish university students [56].	*Sodium*. The Na levels were above those described in a similar S Korean child population (Park et al. 2006) [42], but lower than in Polish university students (Chojnacka, et al. 2010) [56]. Considering that the study population was healthy and showed adequate Na homeostasis, we propose a maximum reference value of **600 µg/g**, which is above the 95th percentile and lower than the 2nd degree atypical value. As the lower value, we propopse the maximum value, after removing atypical and undetected values, such as **3 µg/g**. This range 3-600 **µg/g** includes 96.5% of the total study population.
Mg	**4-100 µg/g**	95th percentile=81.9 µg/g.<br>Percentile 15=5.9 µg/g.<br>Percentile 10=4.2 µg/g.<br>Percentile 5=2.5 µg/g.<br>Including 85% of our population	Similar levels to children in Rome [42] Percentile range 5-95 =7.4-72.9 µg/g.<br>Some higher than S Korean children [44] min value: 3.30 µg/g.	*Magnesium*. The following data are taken under consideration: 1) Mg levels are similar to those reported in a similar population reported in Italy and S Korea (Senofonte et al. 2000; Park et al. 2007) [42,44]. 2) Mg is an element whose homeostasis is well regulated. 3) The atypical values exceed the 95% percentile by excess (maximum 355 µg/g). Published data and Reference Material are showed ranges up to 200 µg/g. Consequently, we suggest considering the 95% percentile as the upper reference value, after being rounded up to **100 µg/g**. The minimum values are reported to lie between 1 and 3 µg/g. We propose the 10% percentile value, rounded off to **4 µg/g**, as the lower reference value.
Al	**< 20 µg/g**	90-95th percentile =18.83-24.78 µg/g, not excluding outliers.<br>Percentile 90-95: 20.8 25.9 µg/g, excluding outliers.<br>X̅A: 7.68 µg/g; Me: 5.98 µg/g, not excluding outliers.	Similar levels to children in Rome [44] P95=20 µg/g; max: 27.6 µg/g and S Korean children [2] max: 25.55 µg/g; X̅A 8.78 µg/g; Me: 8.08 µg/g.<br>8-fold lower than the healthy controls in Arabia and Kuwaiti children with autism [58,59].<br>½-fold lower than the healthy controls in Arabian children with autism [59].	*Aluminium.* The levels reported in the healthy control of studies conducted in Arabia and Kuwait (Fido & Al-Saad 2005; Blaurock-Busch et al. 2012) [58,59] were lower, and higher in Katanga (Elenge et al, 2011) [60] than herein, which were, in turn, similar to those reported in similar populations in Rome and S Korea (Senofonte et al. 2000; Park et al. (2007) [42,44]. In children from Rome, the 95th percentile was 20 µg/g and the maximum value was 27.6 µg/g, while the maximum value in S Korea was 25.55 µg/g. Our 90% percentile, after removing outliers, was 20.8 µg/g, rounded to **20 µg/g** which we propose as the maximum reference value.

			4-fold lower than the healthy college students of Katanga [60].	
K	**2-200 µg/g**	Min-Max: 0.6-155 µg/g, excluding outliers.<br>Percentile 5: 1.8 µg/g, excluding outliers.<br>P97=146 µg/g; P98=169 µg/g, not excluding outliers.<br>Including 97.6% total population of our study.	Similar levels in S Korean children [42].<br>Higher (1x) in Polish students [56].	*Potassium.* The data obtained with a S Korean child population (Park et al. (2007) [42] fell within the same order of magnitude as in our study. The central measures of the Polish study (Chojnacka, et al. 2010) [56] were higher, but with similar dispersion measures. The maximum value after removing outliers was 155 µg/g (6). We suggest taking **160 µg/g** as the maximum reference value. We propose the 5% percentile (1.8 µg/g, Table 7), rounded off to **2 µg/g**, which is lower than the minimum values reported in other studies.
Ca	**20-1000 µg/g**	5-95th percentile = 35-1095 µg/g, excluding outliers.<br>Percentile 5-95: 24-1134 µg/g, not excluding outliers.<br>Including 90% total population of our study.	Similar levels to children in Rome [4].<br>Higher (2x) than S Korean children [42].	*Calcium.* Its levels were similar to those reported in Rome (Senofonte et al. 2000) [44] and about twice those indicated in S Korean children (Park et al. (2007) [42]. We suggest using the 5 and 95th percentiles, respectively rounded off to **25** and **1000 µg/g**, as the lower and upper reference values.
V	**< 0.4 µg/g**	5-95th percentile 5-95: 0.007-0.275 µg/g, excluding outliers.<br>90th percentile 90: 0.237 µg/g, not excluding outliers	Levels 10-fold lower than children in Rome [44].<br>10-fold lower than Polish university students [56].<br>5-fold lower than college students of Katanga [60].	*Vanadium.* In general, our levels were 10-fold lower than those found by Senofonte et al. (2000) [44] in children in Rome, 10-fold lower than those reported by Chojnacka et al. (2010) [56], and 5-fold lower than those indicated for students in Congo in Elenge et al.'s study (2011) [60]. So, a value around the 95% percentile, or of the 1st degree outlier, can be used as a higher reference margen. As the 90% percentile with total N was 0.237, and the 95th percentile was 0.275 µg/g, after eliminating outliers and 0.315 µg/g of ur total sample, we propose using this last, roiunded to **0.3 µg/g** as the higher reference margin.
Cr	**< 0.4 µg/g**	Min-max: 0.006-0.59 µg/g, excluding outliers.<br>Percentile 5-95: 0.005-0.352 µg/g, excluding outliers.<br>95th percentile= 0.357 µg/g, excluding outliers.<br>$\bar{X}$A: 0,215 µg/g, not excluding outliers.	6-fold lower than Spanish children from Catalonia [61].<br>1 or 2-fold lower than Spanish children from Tarragona [8].<br>4-fold lowers than children in Rome [44].<br>2-fold lower than S Korean children [58].<br>4-fold lower than healthy controls in Arabian children with autism [59].<br>15-fold lower than Spanish adult workers in the metal industry from Murcia [62].	*Chromium.* The Cr concentrations found in children from the area of Elche in this study (PC95=0.316 µg/g without outliers or 0,357 µg/g in ur total sample) were considerably lower than those reported by others in Spain were. It is 6-fold lower than in the North-East according to Ferré-Huguet et al. (2009) [61]; ½-fold lower than those indicated by Torrente et al. (2005) [20] in Tarragona province, and 15 times than adults who worked in the metal industry in Murcia Region (Spain) (Gil et al. 2011) [62]. Values are 4-fold lower than in children in Rome [44]; 2-fold lower than in S Koreans [42]; 4 times the healthy controls in Arabia [58] but higher than metal industry worker in Spain [62]. Therefore, we propose using the 95-percentile value, rounded off to **0.4 µg/g**, as the upper reference margin.
Mn	**0.03-1.1 µg/g**	Atypical 2nd grade =0.643 µg/g (including 99%).<br>15th percentile 15= 0.032 µg/g, not excluding outliers.	Lower levels than Spanish children [61], and Italian [44]; S Korean [42], Brazilian [63] and Arabian [59]; [64] children.<br>Minimum concentrations between 0.00 and 0.05 µg/g.	*Manganese.* Its concentrations were lower than those reported in similar child populations of Spain, Italy, Brazil, S Korea and the Arabian Peninsula (Ferré-Huguet et al. (2009; Senofonte et al. 2000; Carneiro et al. 2011; Park et al. 2007; Blaurock-Busch et al. 2011) [42,59,63,64,71] and in an adult population in the Murcia Region (Gil et al. 2011) [62]. Mn is important for physiological functions and its toxicity is related mainly to occupational exposure (which was not the case for our study population). So, we propose using the maximum value in our population **1.1 µg/g** as the higher reference value. The reported minimum concentrations were in the range from 0.00 to 0.05 µg/g (Senofonte et al. 2000; Park et al, 2007; Torrente et al, 2005) [20,42,44]. In our study population, the 15 percentil was 0.032 µg/g in the general population. We suggest rounding it off to **0.030 µg/g** and using this figure as the lower reference margin.

Fe	**2-20 µg/g**	15-95th percentile (5-21 µg/g), excluding outliers (including 80%).	Similar levels to Arabian children [64].<br>Like S Korea [42]: reference value: 7-21 µg/g.<br>2-fold lower than children in Rome [4] and Polish students [56].<br>24-fold lower than Katanga college students [60].	*Iron.* It obtained similar levels to those found in S Korea, whose reference values were 7-21 µg/g (Park et al. 2007) [42], and to those described in Arabia by Blaurock-Busch et al. (2011) [59]. In Italian children, the range of concentrations was similar (Senofonte et al. 2000) [44], but both the mean and median doubled those herein and were similar to the 95th percentile. The levels recorded in Polish students were twice as high and 24-fold higher in Congo (Chojnacka et al. 2010; Elenge et al. 2011) [56,60]. We propose the 5-95th percentiles range, rounded off, as the reference margins: 5-20 µg/g.
Co	**<0.04 µg/g**	85th percentile= 0.026 µg/g, excluding outliers.<br>85th percentile = 0.023 µg/g not excluding outliers.<br>Percentile 90= 0.028 µg/g not excluding outliers.	Similar to S Korea [42]: max: 0.19 µg/g.<br>Similar to Brazilian children: max: 0.027 µg/g.<br>40-fold lower than children in Rome [44].	*Cobalt.* Undetected in 30% of the study population. Its values were 40-fold lower than in children in Rome (Senofonte et al. 2000) [44], similar to those found among S Korean children (Park et al. 2007) [42], twice as high as in Brazilian children (Carneiro et al. (2011), whose maximum value was (0.027 µg/g) and is similar to the 90 percentiles reported herein. We propose using the 95th-percentile value rounded off to **0.04 µg/g**, as the upper reference value.
Ni	**< 0.70 µg/g**	85th percentile =0.468 µg/g not excluding outliers.<br>90th percentile= 0.540 µg/g not excluding outliers.	5-fold lower than children in Rome [44].<br>Lower than Spanish children [61]; [20].<br>Highest mean reported in Spain: 0.48 µg/g [61]; and 0.54 µg/g [20].<br>10-fold lower than Spanish adult workers of the metal industry from Murcia 62].	*Nickel.* The Ni levels in children in Elche were 5-fold lower than in children living in Rome and Tarragona (1998 and 2002) [20,44], and half as low as in 2005 and 2007 in the same area (Senofonte et al. 2000; Ferré-Huguet et al. 2009; Torrente et al. (2005). [20,44,61] Its mean concentration among metalworkers in the Murcia Region (Spain) (Gil et al. 2011) [62] was 10-fold higher than presented herein. The means and medians reported in the child population ranged from 0.48 to 0.54 µg/g. Our 85th percentile in the whole population was 0.468 µg/g and the 95th percentile, after excluding outliers, was 0.647 µg/g. We propose using this figure, after rounding it off to **0.70 µg/g**, as the upper reference value.
Cu	**5-100 µg/g**	65th percentile =98.8 µg/g not excluding outliers.<br>65th percentile =97.9 µg/g excluding outliers.<br>10th percentile =5.4 µg/g, excluding outliers.	Higher than children in Rome: 5-95th percentile= 7.2–82.75 [44].<br>Higher than S Korean children: min- max= 5.82-101.52 µg/g [42].<br>Higher than healthy controls in Arabian [59] and Indian [65] children with autism.	*Copper.* Its levels were 5-fold higher than those reported in Italy and South Korea in similar populations (Senofonte et al. 2000; Park et al. 2007) [42,44]. and than in the healthy controls in the studies published by Blaurock-Busch et al. (2012), and by Priya and Geetha (2011) [59,65]. Our 65th percentile, after eliminating outliers, was similar to the 95th percentile obtained in children from Rome and was practically the same as the maximum concentration reported in S Korea. We propose using our 65th percentile, after rounding off to **100 µg/g**, as the maximum reference level. As the minimum value, we propose employing the 10 percentile (5.4 µg/g, Table 7), rounded off to **5 µg/g**, and taking the 5th percentile value in the children from Rome (7.2 µg/g) and the minimum value found in S Korean children (5.82 µg/g).
Zn	**40-850 µg/g**	10-95th percentile =77-849 µg/g excluding outliers.	7-fold higher than S Korean children [42].<br>2-fold higher than in Turkey [66] and than Polish students [56].<br>9 times higher than Katanga college students [60].<br>3 times higher than healthy controls in Arabian [64] and Indian [65] children with autism.	*Zinc.* Its concentrations in our study population were higher than the data reported in other studies: twice as high as in Turkey (Doğan & Kumbur 2010); 7-fold higher than in S Korea (Park et al. 2007) [42]; twice those in Poland (Chojnacka et al. 2010) [56]; 9-fold higher than in Katanga (Elenge et al. 2011); 3-fold higher than in the Arabian Peninsula (Blaurock-Busch et al. 2011) and India (Priya & Geetha 2011) [56,60,64,65]. Considering that Zn is a relevant oligoelement, and the levels found exceeded those reported, we suggested using the 10-95 percentiles range as reference margins (see Table 7; 77-849 µg/g), after rounding off to **80-850 µg/g**. Within this range, we include 80% of the study population.
As	**<0.1 µg/g**	95th percentile=0.058 µg/g excluding outliers.<br>90th percentile =0.048 µg/g excluding outliers.	3 times lower than children in Rome: Me = 0.06 µg/g [44].<br>1.5 times lower than S Korean children [42].<br>4 times higher than Brazilian children: max= 0.0166 µg/g [63].	*Arsenic.* Our data were 3-fold lower than in Italy and 1.5-fold higher than in S Korea (Senofonte et al. 2000; Park et al. 2007) [42,44], but 4-fold higher than in Brazil (Carneiro et al. 2011) [63]. $\bar{X}_A$ and the mean of S Korean children were 1.5-fold higher than our 95th percentile, with a similar value to the mean value found in Italian children. We propose using the 95% percentile of our total population (0.07 µg/g rounded to **0,1 µg/g** as the maximum reference value.

Se	**0.1-1 μg/g**	Min-max 0.05-1.12 μg/g excluding outliers.<br>5-95th percentile=0.11-0.71 μg/g.	Higher than Brazilian children: min-max = 0.004-0.299 μg/g. [63]<br>Similar than children in Rome [44], and S Korean [42], healthy controls on Arabian [64] and Indian [65] children.	*Selenium.* After removing outliers, the levels coincided with those reported in the studies by Senofonte et al. (2000) and Park et al. (2007) [42,44], and with those indicated for the healthy controls in the studies of Blaurock-Busch et al. (2011), and Priya and Geetha (2010) [59,65]. Its levels in Brazilian adolescents (Carneiro et al. 2011) [63] were considerably lower than in the aforementioned studies. As this element has been described, as a factor to protect against the action of heavy metals like Pb, and the data in this study are comparable to those reported in similar populations, we suggest using **1.00 μg/g** as the upper reference margin. In this figure, we include 97% of the study population. We recommend using the 5 percentile 5 (0.11 μg/g), rounded off to **0.1 μg/g**, as the lower margin.
Sr	**< 5 μg/g**	50th percentile =3.14 μg/g excluding outliers.<br>65th percentile =5.28 μg/g excluding outliers.	2 times higher than children in Rome 3.65 μg/g [44].<br>2 times higher than healthy controls in Arabian children with autism [64].<br>Similar to Polish students [3].	*Strontium.* Its levels were similar to those reported in Polish university students (Chojnacka et al. 2010) [56] and twice as high as in children from Rome and Arabia (Senofonte et al. 2000; Blaurock-Busch et al. 2011) [44,59]. The 95th percentile in children from Rome was below the 65th percentile in Elche and was even lower than $\bar{X}_A$ (4.5 μg/g). We suggest rounding it off to **5 μg/g** and using it as the upper reference limit.
Mo	**<0.1 μg/g**	85th percentile = 0.08 μg/g.<br>90th percentile = 0.09 μg/g.	5 times lower than Italian children [44].<br>1/2 lower than Brazilian and Arabian children [63; 64].<br>Lower than S Korean children [2].<br>Reported average range: 0.06 and 0.09 μg/g.	*Molybdenum.* After removing outliers, values in children from Elche were 5-fold lower than in Italian children, half as low as those figures reported in Brazilian and Arabian children, and slightly lower than in S Korean children. Except for the data found in Italy, where the mean and the median were higher than in the other studies, most authors report means and medians between 0.06 and 0.09 μg/g. The 95% percentile in our population was **0.1 μg/g.** We suggest taking this value as the upper reference margin.
Cd	**<0.1 μg/g**	Min-max= 0.002-0.157 μg/g excluding outliers.<br>5-95th percentile =0.002-0.113 μg/g excluding outliers.<br>Percentile 75=0.046 μg/g	Like another Spanish child population [61].<br>2 times lower than Italy [44], Korea [42] and adult Spanish metal workers [9].<br>2 times lower than healthy controls in Kuwaiti children with autism [58].<br>5 times lower than Katanga students [60].<br>1/2 higher than Brazilian children [63].	*Cadmium.* After removing outliers, the data of this study were similar to those reported in Tarragona (Ferré-Huguet et al. 2009) [61], slightly higher than in Brazil (Carneiro et al. 2011) [63], twice as low as in Murcia Region, S Korea, Italy and Kuwait (Gil et al. 2011; Park et al. 2007; Senofonte et al. 2000; Fido & Al-Saad 2005) [42,44,58,62], and 5-fold lower than in Katanga (Elenge et al. 2011) [60]. We consider that the Cd values in children from Elche were lower than in other studies. Cd is an unhealthy heavy metal, so we suggest taking the 95% percentile value 0.11 μg/g rounded to **0.1 μg/g** as the upper reference margins.
Ba	**<0.9 μg/g**	95th percentile=0.86 μg/g excluding outliers.	Like S Korean children [42].<br>8 times lower than Katanga students [60].<br>5 times lower than Polish students [56].	*Barium.* After removing outliers, its values were similar to those in S Korean children (Park et al. 2007) [42], 8-fold lower than in Congo (Elenge et al. 2011) [60] and 5-fold lower than in Poland (Chojnacka et al. 2010) [56]. Only the work of Park et al. (2007) [42] in S Korea measured this element in children, and its results coincide with ours. The studies by Chojnacka et al. (2010) and Elenge et al. (2011) [56,60] were done in adult populations. As intoxication by barium is not frequent, and is usually accidental and manifested acutely, from the environmental exposure monitoring viewpoint in child populations, we suggest taling the 95th percentile (0.86 μg/g), rounded off to **0.90 μg/g**, as the upper reference margin.
Hg	**<3 μg/g (for Mediterranean and high fish consumer regions)**	Min-max= 0.014-15.66 μg/g excluding outliers.<br>65th percentile =3.34 μg/g excluding outliers.	1/2 higher than in other areas of Spain [19,61,67–70].<br>5 times higher than Flanders, Germany and the USA [71–73].<br>2 times higher than Canada [74].<br>4 times higher than healthy controls in Indian children with autism [65].	*Mercury.* The Hg levels in our study population were very high. A possible explanation for this is the large amount of seafood eaten in Elche, as 98.4% of the study population admitted eating rations of fish/seafood 3 times a week. After removing outliers, $\bar{X}_G$ was half as high as in other Studies conducted in Spain (Garí et al. 2013; Díez et al. 2009; Freire et al. 2010; Batista et al. 1996; Nadal et al. 2005; Ferré-Huguet et al. 2005) [19,61,67–70]. They were twice as high as in Canada (Tian et al. (2011) [74], 4-fold higher than in India children with autism (Priya & Geetha 2010) [74] , 5-fold higher than in Flanders, Germany, the USA, Congo,

	**(<1 µg/g general population)**		5 times higher than Congo and Polish students [56,60].<br>5 times higher than S Koreans [42].<br>3 times lower than the Madeira [75], French Guayana [76] and Faroes [13] Islands.<br>3 times lower than Seychelles Island [14].<br>1/2 times lower than the Philippines and Iran [77,78].<br>5 times lower than Brazil [79].<br>In the range of values and reference values reported in UN from several countries for seafood consumer [80]	Poland and S Korea (Croes et al. 2014; Pesch et al. 2002; McDowell et al. 2004; Elenge et al. 201; Chojnacka et al. 2010 and Park et al. 2007) [42,56,60,71–73]. Our data were one hundred-fold higher than in a rural adult population that we studied in Nicaragua (unpublished data). The levels in children from Elche were lower: half as low as in the Philippines (Drasch et al. 2001) [77] and in Iran (Agah et al. 2010) [78], 3-fold higher than on the Madeira (Murata et al. 2002) [75], French Guiana (Cordier et al. 2002) [76], Faroe (Grandjean et al. 1997) [13] and Seychelle Islands (Myers et al. 2000) [14]. Moreover, they were 5-fold lower than in gold mining areas in infant from Amazon basin in Brazil (Barbosa et al. 1998, 2001) [79],<br>The reference dose (RfD) estimated by the US National Research Council (2000) for the more commonly accepted adverse effect (neurodevelopmental) is of 58 µg/l total Hg in blood from umbilical cord or from 10 µg/g total Hg in mother hair, according to the study of the Faroe Islands (Grandjean et al 1997) [13].<br>The Global Mercury Assessment 2018 in United Nations [80] reported summary results of multiple biomonitoring national programs, including measures in blood, urine and hair, as well as proposed reference values that varie between 1 to 25 µg/g The report conclude that 0.5 and 2 µg/g could be considered background levels for non-consumer and consumer of seafood, respectively. Values in the ranges of 2-5, 5-10, and >10 µg/g could be considered "elevated", "moderately high", and "high" levels, respectively. Although the Hg concentrations in our study population were above the values reported in some populations, should be noted that this work was conducted in a schoolchild population who displayed normal growth and development. Most of this study population lived on the Mediterranean coastline and ate plenty of fish and seafoods. Thus, the reference value needs to be adapted to the characteristics of the area and, as with all the elements in the present work, the selection criteria of the reference values are statistical and not clinical. We propose taking the 65 percentile (3.36 µg/g), rounded off to **3 µg/g**, as the maximum reference value of the population in this geographic area and in similar regions with diets that include a lot of fish. However, the value recommended of **< 2 µg/g** or indeed **< 1 µg/g** must continue to be considered the reference value for the world general population, which does not eat so many fish and seafoods.
Tl	**<0.001 µg/g**	95th percentile: 0.001 µg/g	No data evaluated.	*Thallium*. Tl was detected only in 21% of samples, and at a maximum concentration of 0.006 µg/g, but 95% of samples presented levels below 0.001 µg/g. It can thus be stated that it is not usuall to find this element in hair samples, but it is possible to find levels below **0.001 µg/g**. Therefore, we suggest taking this value as the upper reference margin.
Pb	**<2 µg/g**	$\bar{X}$G=0.95 µg/g excluding outliers.<br>$\bar{X}$A=1.16 µg/g excluding outliers.<br>Me= 1.14 µg/g excluding outliers.<br>50th percentile =1.14 µg/g excluding outliers.<br>65th percentile =1.79 µg/g excluding outliers.<br>65th percentile =1.92 µg/g not excluding outliers.	Italian children [81]: $\bar{X}$A= 0.74 µg/g.<br>Another area of the Spanish arithmetic mean range: 0.56-0.73 µg/g.<br>2-fold lower than Sestu (Italy) children: $\bar{X}$G=3.19 µg/g [81].<br>9-fold lower than Portucoso (Italy) children: $\bar{X}$G=11.85 µg/g [81].<br>Similar and 1/2 higher than children from Spain [20,61]<br>German children: $\bar{X}$G = 1.61 µg/g [82].<br>.	*Lead*. Most published reports and studies conclude that blood is the best matrix to control exposure to Pb. There is no known safe blood lead concentration. However, it is known that, as lead exposure increases, the range and severity of symptoms and effects increases. Sanna et al. (2011) [81] described a significant correlation between levels of Pb in hair (PbH) and blood (PbB) when PbB concentrations were ≥ 5 µg/dl. These authors concluded that it is necessary to check these findings by conducting studies with a larger population sample, and that hair and urine matrices do not substitute the blood value. Yet there is one interesting piece of evidence: in one of the three populations included (Perdasdefogu, n=53), the $\bar{X}_A$ of PbB was 5.30 µg/dl and the $\bar{X}_A$ of PbH was 0.74 µg/g. Although the PbB-PbH correlation was not completely demonstrated, the $\bar{X}_A$ of PbH in Sanna et al.'s study (2011) [81] is helpful as a starting point to select a value to be proposed as a reference upper limit. Our $\bar{X}_G$ (0.947 µg/g), after excluding atypical values, is only slightly higher, so it could be a good candidate, and it is also a similar value to the $\bar{X}_A$ reported in children in Tarragona in 2002 (Ferré-Huguet et al. 2009) [61]. Among the healthy controls in the work by

				Priya and Geetha (2011) [65] conducted in India, $\bar{X}_A$ was 1.56 µg/g, which is slightly lower than our 65 percentile (1.79 µg/g), after excluding atypical values. A German survey levels around $\bar{X}G$ = 1.61 µg/g were reported [82].Our 65 percentile, without excluding atypical values, is 1.92 µg/g (Table 4). If we round it off to 2 µg/g, we obtain a practical figure that includes 65% of the study population without excluding the extreme values, and practically 75% of the population when we do exclude them. We therefore propose taking the **2 µg/g** value as the upper limit of the Pb references in hair.
Bi	**<0.03 µg/g**	95th percentile=0.024 µg/g excluding outliers.	Lower order of magnitude than S Korean children [2].	*Bismuth.* This element was not detected in 41 cases (10% of the whole population). Eliminating the outliers left the values around one order of magnitude below those described by Park et al. (2007) [42] in S Korea, the only study that reported this element. Hence from a statistical viewpoint, we suggest using the 95th percentile value (0.024 µg/g), rounded off to **0.03 µg/g**, as the upper reference margin.
Ag	**<0.01 µg/g**	99.8% of samples: ≤0.010 µg/g.	Lower than Katanga and Polish students [6,3].	*Silver.* Silver values were lower, by one order of magnitude, than those reported in healthy university students in Congo and Poland (Elenge et al. 2011; Chojnacka et al. 2010) [56,60]. Silver was detected only in 28% of samples, and in 99.8% of cases, its levels were under **0.010 µg/g**. We propose taking this figure as the maximum reference value.
Au	**<0.2 µg/g**	95th percentile=0.17 µg/g.	No data evaluated.	*Gold.* Au went undetected in 78% of cases. No similar studies were found. It was detected in 22% of the samples, and although we did not expect to find this element in hair, we propose the 95% percentile value to be the maximum limit, rounded off to **0.2 µg/g**, to make the practical application of the table of reference values easier.
U	**<0.001**	Undetected	No data evaluated.	*Uranium.* U was not detected in any sample. It can thus be stated that it is not normal to find this element in hair samples, but it is possible to find levels below **0.001 µg/g**. Therefore, we suggest taking this value as the upper reference margin.

References

2. Otim, E.O.; Chen, R.; Otim, O. Applying multivariate analysis to characterize waragi spirits from Acoli, Uganda, by their metal contents. *Heliyon* **2019**, *5*, e01417. https://doi.org/10.1016/j.heliyon.2019.e01417.
3. Kumari, S.; Jain, M.K.; Elumalai, S.P. Assessment of Pollution and Health Risks of Heavy Metals in Particulate Matter and Road Dust Along the Road Network of Dhanbad, India. *J. Health Pollut.* **2021**, *11*, 210305. https://doi.org/10.5696/2156-9614-11.29.210305.
4. Zoroddu, M.A.; Aashet, J.; Crisponi, G.; Medici, S.; Peana, M.; Nurchi, V.M. The essential metals for humans: A brief overview. *J. Inorg. Biochem.* **2019**, *195*, 120–129. https://doi.org/10.1016/j.jinorgbio.2019.03.013.
6. Scheinberg, I.H.; Sternlieb, I. Wilson disease and idiopathic copper toxicosis. *Am. J. Clin. Nutr.* **1996**, *63*, 842S–845S.
8. Cernichiari, E.; Myers, G.J.; Ballatori, N.; Zareba, G.; Vyas, J.; Clarkson, T. The biological monitoring of prenatal exposure to methylmercury. *Neurotoxicology.* **2007**, *28*, 1015–1022.
9. Cernichiari, E.; Toribara, T.Y.; Liang, L.; Marsh, D.O.; Berlin, M.W.; Myers, G.J.; Cox, C.; Shamlaye, C.F.; Choisy, O.; Davidson P The biological monitoring of mercury in the Seychelles study. *Neurotoxicology* **1995**, *16*, 613–628.
13. Grandjean, P.; Weihe, P.; White, R.; Bebes, F.; Araki, S.; Yokoyama, K.; Murata, K.; Sorensen, N.; Dahl, R.; Jorgensen, P. Cognitive déficit in 7 year-old children with prenatal exposure to methylmercury. *Neurotoxicology Teratol.* **1997**, *19*, 417–428.
14. Myers, G.J.; Davidson, P.W.; Palumbo, D.; Shamlaye, C.; Cox, C.; Cernichiari, E.; Clarkson TW Secondary Analisis from the Seycheles Child Developmente Study: The Chile Behavior Ckecklist. *Environ. Res.* **2000**, *84*, 12–19.
19. Nadal, M.; Bocio, A.; Schuhmacher, M.; Domingo, J.L. Monitoring metals in the population living in the vicinity of a hazardous waste incinerator: Levels in hair of school children. *Biol. Trace Elem. Res.* **2005**, *104*, 203–213.
20. Torrente, M.; Colomina, M.-T.; Domingo, J. Metal Concentrations in Hair and Cognitive Assessment in an Adolescent Population. *Biol. Trace Elem. Res.* **2005**, *104*, 215.
42. Park, H.-S.; Shin, K.-O.; Kim, J.-S. Assessment of Reference Values for Hair Minerals of Korean Preschool Children. *Biol. Trace Elem. Res.* **2007**, *116*, 119–130.
44. Rodrigues, J.L.G.; Araújo, C.F.S.; Dos Santos, N.R.; Bandeira, M.J.; Anjos, A.L.S.; Carvalho, C.F.; Lima, C.S.; Abreu, J.N.S.; Mergler, D.; Menezes-Filho, J.A. Airborne manganese exposure and neurobehavior in school-aged children living near a ferro-manganese alloy plant. *Environ. Res.* **2018**, *167*, 66–77. https://doi.org/10.1016/j.envres.2018.07.007.

56. Chojnacka, K.; Zielińska, A.; Gorecka, H.; Dobrzański, Z.; Gorecki, H. Reference values for hair minerals of Polish students. *Environ. Toxicol. Pharmacol.* **2010**, *29*, 314–319.
58. Fido, A.; Al-Saad, S. Toxic trace elements in the hair of children with autism. *Autism* **2005**, *9*, 290–298.
59. Blaurock-Busch, E.; Amin, O.R.; Rabah, T. Heavy metals and trace elements in hair and urine of a sample of arab children with autistic spectrum disorder. *Maedica (Bucur).* **2011**, *6*, 247–57.
60. Elenge, M.M.; Aubry, J.C.; Jacob, L.; De B.rouwer, C. Heavy metal in hair samples of 109 non-industrial (miners) population in Katanga. *Sante* **2011**, *21*, 41–46.
61. Ferré-Huguet, N.; Nadal, M.; Schuhmacher, M.; Domingo, J.L. Monitoring Metals in Blood and Hair of the Population Living Near a Hazardous Waste Incinerator: Temporal Trend. *Biol. Trace Elem. Res.* **2009**, *128*, 191–199.
62. Gil, F.; Hernández, A.F.; Márquez, C.; Femia, P.; Olmedo, P.; López-Guarnido, O.; Pla, A. Biomonitorization of cadmium, chromium, manganese, nickel and lead in whole blood, urine, axillary hair and saliva in an occupationally exposed population. *Sci. Total Environ.* **2011**, *409*, 1172–1180.
63. Carneiro, M.F.; Moresco, M.B.; Chagas, G.R.; de Oliveira Souza, V.C.; Rhoden, C.R.; Barbosa, F., Jr. Assessment of Trace Elements in Scalp Hair of a Young Urban Population in Brazil. *Biol. Trace Elem. Res.* **2011**, *143*, 815–824.
64. Blaurock-Busch, E.; Amin, O.R.; Dessoki, H.H.; Rabah, T. Toxic Metals and Essential Elements in Hair and Severity of Symptoms among Children with Autism. *Maedica (Buchar)* **2012**, *7*, 38–48.
65. Priya, L.; Geetha, A. Level of Trace Elements (Copper, Zinc, Magnesium and Selenium) and Toxic Elements (Lead and Mercury) in the Hair and Nail of Children with Autism. *Biol. Trace Elem. Res.* **2011**, *142*, 148–158.
66. Doğan-Sağlamtimur, N.; Kumbur, H. Metals (Hg, Pb, Cu, and Zn) Bioaccumulation in Sediment, Fish, and Human Scalp Hair: A Case Study from the City of Mersin Along the Southern Coast of Turkey. *Biol. Trace Elem. Res.* **2010**, *136*, 55–70.
67. Garí, M.; Grimalt, J.O.; Torrent, M.; Sunyer, J. Influence of socio-demographic and diet determinants on the levels of mercury in preschool children from a Mediterranean island. *Environ. Pollut.* **2013**, *182*, 291–298.
68. Díez, S.; Delgado, S.; Aguilera, I.; Astray, J.; Pérez-Gómez, B.; Torrent, M.; Sunyer, J.; Bayona, J. Prenatal and Early Childhood Exposure to Mercury and Methylmercury in Spain, a High-Fish-Consumer Country. *Arch. Environ. Contam Ina. Toxicolpgy* **2009**, *56*, 615–622.
69. Freire, C.; Ramos, R.; Lopez-Espinosa, M.J.; Díez, S.; Vioque, J.; Ballester, F.; Fernández, M.F. Hair mercury levels, fish consumption, and cognitive development in preschool children from Granada, Spain. *Environ. Res.* **2010**, *110*, 96–104.
70. Batista, J.; Schuhmacher, M.; Domingo, J.L.; Corbella, J. Mercury in hair for a child population from Tarragona Province, Spain. *Sci. Total Environ.* **1996**, *193*, 143–148
71. Croes, K.; De Coster, S.; De Galan, S.; Morrens, B.; Loots, I.; Van de Mieroop, E.; Nelen, V.; Isabelle Sioen, I.; Bruckers, L.; Nawrot, T.; Colles, A.; Den Hond, E.; Schoeters, G.; Larebekeb, N.; Baeyens, W.; Gao, Y. Health effects in the Flemish population in relation to low levels ofmercury exposure: From organ to transcriptome level. *Int. J. Hyg. Environ. Health* **2014**, *217*, 239–247.
72. Pesch, A.; Wilhelm, M.; Rostek, U.; Schmitz, N.; Weishoff-Houben, M.; Ranft, U.; Idel, H. Mercury concentrations in urine, scalp hair, and saliva in children from Germany. *J. Expo. Anal. Environ. Epidemiol.* **2002**, *12*, 252 – 258.
73. McDowell, M.; Dillon, C.; Osterloh, J.; Bolger, P.M.; Pellizzari, E.; Fernando, R.; Montes de Oca, R.; Schober, S.; Sinks, T.; Jones, R.; Mahaffey K. Hair Mercury Levels in U.S. Children and Women of Childbearing Age: Reference Range Data from NHANES 1999–2000. *Environ. Health Perspect.* **2004**, *112*, 1165–1171.
74. Tian, W.; Egeland, G.; Sobol, I.; Chan, H. Mercury hair concentrations and dietary exposure among Inuit preschool children in Nunavut, Canada. *Environ. Int.* **2011**, *37*, 42–48.
75. Murata, K., Budtz-Jørgensen, E.; and Grandjean, P. Benchmark Dose Calculations for Methylmercury- Associated Delays on Evoked Potential Latencies in Two Cohorts of Children. *Risk Anal.* **2002**, *22*, 465–474
76. Cordier, S.; Garel, M.; Mandereau, L.; Morcel, H.; Doineau, P.; Gosme-Seguret, S.; Josse, D.; White, R.; and Amiel-Tison, C. Neurodevelopmental Investigations among Methylmercury-Exposed Children in French Guiana. *Environ. Res. Sect. A* **2002**, *89*, 1–11.
77. Drasch, G.; Böse-O'Reilly, S.; Beinhoff, C.; Roider, G.; Maydl, S. The Mt. Diwata study on the Philippines 1999 - assessing mercury intoxication of the population by small scale gold mining. *Sci. Total Environ.* **2001**, *267*, 151–168.
78. Agah, H.; Leermakers, M.; Gao, Y.; Fatemi SMR, Katal, M.M.; Baeyens, W.; Elskens, M. Mercury accumulation in fish species from the Persian Gulf and in human hair from fishermen. *Environ. Monit. Assess.* **2010**, *169*, 203–216.
79. Barbosa, A.C.; Jardim, W.; Dórea, J.G.; Fosberg, B.; Souza, J. Hair mercury speciation as a function of gender, age, and body mass index in inhabitants of the Negro River basin, Amazon, Brazil. *Arch. Environ. Contam Toxicol.* **2001**, *40*, 439–44.
80. (*) UNE (2019) Global Mercury Assessment Technical Background Report 2018. Published 2019. UN Environment. Programme (Geneva, Switzerland). ISBN – 978-82-7971-108-7. https://www.unep.org/resources/publication/global-mercury-assessment-technical-background-report (accessed 28-04-2023)
81. Sanna, E.; De Micco, A.; Vallascas, E. Evaluation of Association between Biomarkers of Lead Exposure in Sardinian Children (Italy). *Biol. Trace Elem. Res.* **2011**, *143*, 1383–1392.
82. Seifert, B.; Becker, K.; Helm, D.; Krause, C.; Schulz, C.; Seiwert, M. The German Environmental Survey 1990/1992 (GerES II): reference concentrations of selected environmental pollutants in blood, urine, hair, house dust, drinking water and indoor air. *J. Expo. Anal. Environ. Epidemiol.* **2000**, *10*, 552–565.
