# Supplementary material for: Reference Values on Children’s Hair for 28 Elements (Heavy Metals and Essential Elements) Based on a Pilot Study in a Representative Non-Contaminated Local Area
Source: Int J Mol Sci. 2023 May 1;24(9):8127. doi: 10.3390/ijms24098127 (PMC10179086; doi:10.3390/ijms24098127)
Supplement: Supplementary file 1 [file ijms-24-08127-s001.zip › Tables S1-S4.pdf]

# TABLES SUPPLEMENTARY MATERIAL

## Table S1. The ICP analysis standard concentration range.

| Elem. | m/z | Certified concentrations | Concentrations range of standard dilutions | ISTD m/z | ISTD concentration |
|---|---|---|---|---|---|
| Be | 9 | 20 µg/l | 0.018-17.82 µg/l | Sc 45 | 90.9 µg/l |
| B | 11 | 102 µg/l | 0.091-90.87 µg/l | Sc 45 | 90.9 µg/l |
| Na | 23 | 7271 µg/l | 6.478-6478 µg/l | Sc 45 | 90.9 µg/l |
| Mg | 24 | 14946 µg/l | 13.32-13300 µg/l | Sc 45 | 90.9 µg/l |
| Al | 27 | 1000 mg/l | 9.145-9145 µg/l | Sc 45 | 90.9 µg/l |
| K | 39 | 2703 µg/l | 2.408-2408 µg/l | Sc 45 | 90.9 µg/l |
| Ca | 43 | 35381 µg/l | 31.52-31500 µg/l | Sc 45 | 90.9 µg/l |
| V | 51 | 50.6 µg/l | 0.045-45.08 µg/l | Sc 45 | 90.9 µg/l |
| Cr | 53 | 20.3 µg/l | 0.018-18.09 µg/l | Sc 45 | 90.9 µg/l |
| Mn | 55 | 30.2 µg/l | 0.027-26.91 µg/l | Sc 45 | 90.9 µg/l |
| Fe | 56 | 100 µg/l | 0.089-89.09 µg/l | Sc 45 | 90.9 µg/l |
| Co | 59 | 25 µg/l | 0.022-22.27 µg/l | Sc 45 | 90.9 µg/l |
| Ni | 60 | 50.3 µg/l | 0.045-44.81 µg/l | Sc 45 | 90.9 µg/l |
| Cu | 63 | 20.5 µg/l | 0.018-18.26 µg/l | Sc 45 | 90.9 µg/l |
| Zn | 66 | 49 µg/l | 0.044-43.66 µg/l | Sc 45 | 90.9 µg/l |
| As | 75 | 50.6 µg/l | 0.045-45.08 µg/l | Y 89 | 90.9 µg/l |
| Se | 82 | 10.4 µg/l | 0.009-9.265 µg/l | Y 89 | 90.9 µg/l |
| Sr | 88 | 106 µg/l | 0.094-94.44 µg/l | Y 89 | 90.9 µg/l |
| Mo | 95 | 104 µg/l | 0.093-92.66 µg/l | Y 89 | 90.9 µg/l |
| Ag | 107 | --- | --- | Y 89 | 90.9 µg/l |
| Cd | 111 | 19.8 µg/l | 0.018-17.64 µg/l | Y 89 | 90.9 µg/l |
| Ba | 137 | 50.6 µg/l | 0.045-45.08 µg/l | Re 185 | 90.9 µg/l |
| Au | 197 | --- | --- | Re 185 | 90.9 µg/l |
| Hg | 202 | 10 mg/l | 0.091-90.91 µg/l | Re 185 | 90.9 µg/l |
| Tl | 205 | 10.4 µg/l | 0.009-9.265 µg/l | Re 185 | 90.9 µg/l |
| Pb | 208 | 25.5 µg/l | 0.023-22.72 µg/l | Re 185 | 90.9 µg/l |
| Bi | 209 | 10.5 µg/l | 0.009-9.355 µg/l | Re 185 | 90.9 µg/l |
| U | 238 | --- | --- | Re 185 | 90.9 µg/l |
| Cu* | 63 | 8.658 µg/l | 49.15-7864 µg/l | Sc 45 | 90.9 µg/l |
| Zn* | 66 | 58.600 µg/l | 333-53300 µg/l | Sc 45 | 90.9 µg/l |

* For Cu and Zn, it was necessary to make three standards to higher concentrations using salts of Zinc sulfate 7-hydrate and Copper sulfate pentahydrate PANREAC® (Spain).

**Table S2. Upper outlier values (µ/g) using the quartiles* technique.**

| Element | Q1 | Q2 | Q3 | IQR | 1st grade | 2nd grade** |
|---|---|---|---|---|---|---|
| **Be** | 0 | 0 | 0.001 | 0.001 | 0.003 | 0.004 |
| **B** | 1.17 | 2.33 | 3.59 | 2.42 | 7.22 | 10.85 |
| **Na** | 44.2 | 97.7 | 189.3 | 145.1 | 406.9 | 624.5 |
| **Mg** | 8.6 | 18.7 | 39.9 | 31.3 | 86.9 | 133.9 |
| **Al** | 0.9 | 5.98 | 11.94 | 11.04 | 28.5 | 45.06 |
| **K** | 7.3 | 21 | 45.3 | 38 | 102.4 | 159.4 |
| **Ca** | 127.4 | 278.9 | 567 | 439.6 | 1226.5 | 1885.9 |
| **V** | 0.032 | 0.077 | 0.151 | 0.119 | 0.33 | 0.508 |
| **Cr** | 0.024 | 0.135 | 0.199 | 0.175 | 0.462 | 0.724 |
| **Mn** | 0.067 | 0.119 | 0.211 | 0.144 | 0.427 | 0.643 |
| **Fe** | 5.4 | 8.78 | 12.72 | 7.32 | 23.69 | 34.67 |
| **Co** | 0.00 | 0.006 | 0.016 | 0.016 | 0.04 | 0.064 |
| **Ni** | 0.062 | 0.216 | 0.365 | 0.303 | 0.82 | 1.274 |
| **Cu** | 16.2 | 67.2 | 142.2 | 126 | 331.2 | 520.1 |
| **Zn** | 285.9 | 485.5 | 634.6 | 348.7 | 1157.6 | 1680.7 |
| **As** | 0.011 | 0.02 | 0.033 | 0.022 | 0.066 | 0.099 |
| **Se** | 0.36 | 0.48 | 0.56 | 0.2 | 0.87 | 1.17 |
| **Sr** | 1.24 | 3.14 | 7 | 5.77 | 15.65 | 24.3 |
| **Mo** | 0.02 | 0.039 | 0.063 | 0.043 | 0.128 | 0.192 |
| **Cd** | 0.013 | 0.025 | 0.049 | 0.036 | 0.103 | 0.157 |
| **Ba** | 0.12 | 0.289 | 0.499 | 0.379 | 1.068 | 1.636 |
| **Hg** | 0.742 | 2.087 | 4.671 | 3.929 | 10.565 | 16.458 |
| **Tl** | 0.00 | 0.00 | 0.00 | 0.00 | 0.00 | 0.00 |
| **Pb** | 0.5 | 1.167 | 2.537 | 2.037 | 5.593 | 8.648 |
| **Bi** | 0.002 | 0.005 | 0.012 | 0.01 | 0.027 | 0.042 |
| **Ag** | 0.00 | 0.00 | 0.001 | 0.001 | 0.003 | 0.004 |
| **Au** | 0.00 | 0.00 | 0.00 | 0.00 | 0.00 | 0.00 |
| **U** | 0.00 | 0.00 | 0.00 | 0.00 | 0.00 | 0.00 |

* Values are expressed as µg/g

** Upper outlier of the first grade = Q3 + (IQR x 1.5); upper outlier of the second grade: Q3 + (IQR x 3). IQR= Q3-Q1

**Table S3. General statistics after eliminating values below the LOD and outlier excess of the second degree*.**

| Elem. | N** | LOD | Min-Max. | $\bar{X}_G$ | $\bar{X}_A \pm SD$ | CI 95% ($\bar{X}_A$) | Me | $\Upsilon_1$ | $\Upsilon_2$ | K-S (*p*) |
|---|---|---|---|---|---|---|---|---|---|---|
| B | 376 | 0.08 | 0.08-9.46 | 1.96 | 2.71±1.72 | 3.08-3.37 | 2.46 | 1 | 1 | <0.01 |
| Na | 409 | 2.8 | 3-623 | 83 | 137±127 | 125-149 | 98 | 1.5 | 2 | <0.01 |
| Mg | 360 | 0.1 | 0.1-43.4 | 5.3 | 8.9±7.7 | 8.1-9.7 | 7.2 | 1 | 1.6 | <0.01 |
| Al | 348 | 0.4 | 0.4-43.4 | 5.9 | 9.2±7.7 | 8.4-10 | 7.5 | 1 | 1.6 | <0.01 |
| K | 384 | 0.56 | 0.6-155 | 19 | 32±30 | 29-35 | 22 | 1.5 | 2 | <0.01 |
| Ca | 403 | 0.8 | 9-1465 | 254 | 384±318 | 352-415 | 285 | 1 | 1 | <0.01 |
| V | 409 | 0.0004 | 0.001-0.46 | 0.06 | 0.10±0.09 | 0.09-0.11 | 0.07 | 1 | 1.5 | <0.01 |
| Cr | 350 | 0.005 | 0.006-0.59 | 0.12 | 0.16±0.107 | 0.15-0.17 | 0.155 | 1 | 2 | <0.01 |
| Mn | 400 | 0.004 | 0.004-0.64 | 0.11 | 0.16±0.124 | 0.15-0.17 | 0.124 | 1 | 1.4 | <0.01 |
| Fe | 375 | 1.2 | 1.1-32.7 | 8.3 | 10.0±5.5 | 09-11 | 9.2 | 0.9 | 1.3 | <0.01 |
| Co | 280 | 0.001 | 0.001-0.063 | 0.01 | 0.014±0.011 | 0.013-0.015 | 0.012 | 1.4 | 2 | <0.01 |
| Ni | 385 | 0.005 | 0.01-1.26 | 0.16 | 0.26±0.21 | 0.24-0.28 | 0.22 | 1 | 3 | <0.01 |
| Cu | 414 | 0.05 | 0.7-515 | 47 | 98±105 | 88-108 | 66 | 1.7 | 3 | <0.01 |
| Zn | 417 | 0.13 | 10-1422 | 358 | 477±277 | 450-504 | 484 | 0.4 | 0 | <0.05 |
| As | 376 | 0.003 | 0.003- 0.099 | 0.019 | 0.024±0.017 | 0.022-0.026 | 0.02 | 1.4 | 2 | <0.01 |
| Se | 377 | 0.05 | 0.05-1.12 | 0.42 | 0.46±0.17 | 0.45-0.48 | 0.49 | -0.3 | 1 | <0.01 |
| Sr | 415 | 0.02 | 0.04-19.6 | 2.7 | 4.5±4.0 | 4.2-4.9 | 3.1 | 1 | 0.4 | <0.01 |
| Mo | 394 | 0.001 | 0.002-0.162 | 0.032 | 0.045±0.031 | 0.041-0.048 | 0.04 | 0.9 | 0.9 | <0.01 |
| Cd | 389 | 0.002 | 0.002-0.157 | 0.024 | 0.036±0.033 | 0.033-0.039 | 0.025 | 1.6 | 2 | <0.01 |
| Ba | 413 | 0.002 | 0.004-1.59 | 0.22 | 0.35±0.28 | 0.32-0.37 | 0.29 | 1.3 | 2 | <0.01 |
| Hg | 411 | 0.003 | 0.014-15.66 | 1.56 | 3.12±3.16 | 2.84-3.45 | 2.04 | 1 | 2 | <0.01 |
| Pb | 405 | 0.004 | 0.032-8.588 | 0.947 | 1.692±1.708 | 1.525-1.859 | 1.138 | 2 | 3 | <0.01 |
| Bi | 341 | 0.0002 | 0.001-0.040 | 0.005 | 0.008±0.008 | 0.007-0.009 | 0.005 | 2 | 4 | <0.01 |

* All values as µg/g; LOD: limit of detection; $\bar{X}_A$: arithmetic mean; $\bar{X}_{G:}$ geometric mean; SD: standard deviations; Me: median; $\Upsilon_{1:}$ asymmetry; $\Upsilon_{2:}$ kurtosis; KS: Kolmogorov-Smirnov test.

** “N” the number of individuals remaining after removing the cases with values below the LOD and outliers excess for 2nd degree)

**Table S4. Percentiles after eliminating values below the LOD and outliers excess of second grade*.**

| | N** | LOD | **5** | 10 | 15 | 25 | **50** | 65 | 75 | 80 | 85 | 90 | **95** |
|---|---|---|---|---|---|---|---|---|---|---|---|---|---|
| B | 376 | 0.08 | **0.17** | 0.31 | 0.87 | 1.70 | **2.46** | 3.05 | 3.73 | 4.03 | 4.41 | 4.93 | **6.12** |
| Na | 409 | 2.8 | **8.5** | 13.9 | 25.8 | 45.5 | **97.9** | 146.2 | 189.1 | 215.4 | 246.8 | 320.7 | **428.7** |
| Mg | 360 | 0.1 | **0.4** | 0.7 | 1.3 | 3.2 | **7.2** | 10.4 | 12.8 | 13.9 | 15.9 | 20.4 | **25.8** |
| Al | 348 | 0.4 | **0.6** | 0.9 | 1.5 | 3.3 | **7.5** | 10.7 | 13.0 | 14.1 | 16.0 | 20.8 | **25.9** |
| K | 384 | 0.56 | **1.8** | 3.6 | 4.9 | 9.8 | **22.0** | 33.3 | 44.5 | 55.0 | 63.4 | 76.4 | **92.3** |
| Ca | 403 | 0.8 | **35** | 56 | 83 | 139 | **285** | 422 | 563 | 642 | 709 | 854 | **1095** |
| V | 409 | 0.0004 | **0.007** | 0.015 | 0.021 | 0.032 | **0.074** | 0.117 | 0.143 | 0.162 | 0.186 | 0.220 | **0.275** |
| Cr | 350 | 0.005 | **0.012** | 0.020 | 0.037 | 0.101 | **0.155** | 0.185 | 0.212 | 0.229 | 0.248 | 0.284 | **0.352** |
| Mn | 400 | 0.004 | **0.011** | 0.025 | 0.043 | 0.075 | **0.124** | 0.166 | 0.213 | 0.251 | 0.291 | 0.336 | **0.432** |
| Fe | 375 | 1.2 | **1.7** | 3.0 | 4.9 | 6.2 | **9.2** | 11.4 | 12.9 | 14.0 | 15.2 | 16.9 | **20.9** |
| Co | 280 | 0.001 | **0.001** | 0.002 | 0.003 | 0.006 | **0.012** | 0.016 | 0.019 | 0.022 | 0.026 | 0.030 | **0.038** |
| Ni | 385 | 0.005 | **0.020** | 0.020 | 0.040 | 0.090 | **0.220** | 0.290 | 0.365 | 0.400 | 0.451 | 0.540 | **0.647** |
| Cu | 414 | 0.05 | **3.3** | 5.4 | 7.7 | 15.6 | **65.7** | 97.9 | 138.0 | 159.6 | 196.9 | 250.7 | **333.0** |
| Zn | 417 | 0.13 | **39** | 77 | 131 | 285 | **484** | 567 | 633 | 675 | 754 | 849 | **969** |
| As | 376 | 0.003 | **0.005** | 0.007 | 0.010 | 0.012 | **0.020** | 0.026 | 0.032 | 0.036 | 0.040 | 0.048 | **0.058** |
| Se | 377 | 0.05 | **0.11** | 0.17 | 0.27 | 0.41 | **0.49** | 0.53 | 0.56 | 0.58 | 0.60 | 0.65 | **0.71** |
| Sr | 415 | 0.02 | **0.31** | 0.53 | 0.70 | 1.26 | **3.14** | 5.28 | 6.99 | 8.01 | 8.96 | 10.89 | **13.21** |
| Mo | 394 | 0.001 | **0.004** | 0,006 | 0.01 | 0.02 | **0.04** | 0.05 | 0.06 | 0.07 | 0.08 | 0.09 | **0.10** |
| Cd | 389 | 0.002 | **0.003** | 0.006 | 0.008 | 0.014 | **0.025** | 0.036 | 0.046 | 0.056 | 0.069 | 0.082 | **0.113** |
| Ba | 413 | 0.002 | **0.02** | 0.04 | 0.06 | 0.12 | **0.29** | 0.41 | 0.50 | 0.57 | 0.64 | 0.71 | **0.86** |
| Hg | 411 | 0.003 | **0.08** | 0.19 | 0.30 | 0.72 | **2.05** | 3.34 | 4.44 | 5.42 | 6.70 | 7.95 | **9.42** |
| Pb | 405 | 0.004 | **0.09** | 0.14 | 0.21 | 0.49 | **1.14** | 1.79 | 2.38 | 2.83 | 3.36 | 3.86 | **5.24** |
| Bi | 341 | 0.0002 | **0.001** | 0.001 | 0.002 | 0.002 | **0.005** | 0.007 | 0.010 | 0.012 | 0.015 | 0.018 | **0.024** |

* All values as µg/g.
** Column "N" shows the number of individuals remaining after removing the cases with values below the LOD and outliers (excess for 2nd degree).
